# Integration of multi-omics data reveals cis-regulatory variants that are associated with phenotypic differentiation of eastern from western pigs

**DOI:** 10.1186/s12711-022-00754-2

**Published:** 2022-09-14

**Authors:** Yuwen Liu, Yang Fu, Yalan Yang, Guoqiang Yi, Jinmin Lian, Bingkun Xie, Yilong Yao, Muya Chen, Yongchao Niu, Lei Liu, Liyuan Wang, Yongsheng Zhang, Xinhao Fan, Yijie Tang, Pengxiang Yuan, Min Zhu, Qiaowei Li, Song Zhang, Yun Chen, Binhu Wang, Jieyu He, Dan Lu, Ivan Liachko, Shawn T. Sullivan, Bin Pang, Yaoqing Chen, Xin He, Kui Li, Zhonglin Tang

**Affiliations:** 1 Shenzhen Branch, Guangdong Laboratory for Lingnan Modern Agriculture, Key Laboratory of Livestock and Poultry Multi-Omics of MARA, Agricultural Genomics Institute at Shenzhen, Chinese Academy of Agricultural Sciences, 518124 Shenzhen, China; 2 Kunpeng Institute of Modern Agriculture at Foshan, Chinese Academy of Agricultural Sciences, Foshan, 528226 China; 3 Innovation Group of Pig Genome Design and Breeding, Research Centre for Animal Genome, Agricultural Genomics Institute at Shenzhen, Chinese Academy of Agricultural Sciences, Shenzhen, 518124 China; 4 Biozeron Shenzhen, Inc., Shenzhen, 518081 China; 5 Guangxi Key Laboratory of Livestock Genetic Improvement, Guangxi Institute of Animal Science, Nanning, 530001 China; 6 Phase Genomics, Inc., Seattle, USA; 7 Kobilka Institute of Innovative Drug Discovery, School of Life and Health Sciences, Chinese University of Hong Kong, Shenzhen, 518172 Guangdong China; 8 School of Public Health (Shenzhen), Sun Yat-Sen University, Shenzhen, China; 9 Department of Human Genetics, The University of Chicago, Chicago, IL 60637 USA; 10 Institute of Animal Sciences, Chinese Academy of Agricultural Sciences, Beijing, 100193 China; 11 Research Centre of Animal Nutritional Genomics, State Key Laboratory of Animal Nutrition, Institute of Animal Sciences, Chinese Academy of Agricultural Sciences, Shenzhen, 518124 China; 12 Resource Development of Bama Xiang Pig, Guangxi Engineering Research Center, Bama, 547500 Guangxi China

## Abstract

**Background:**

The genetic mechanisms that underlie phenotypic differentiation in breeding animals have important implications in evolutionary biology and agriculture. However, the contribution of cis-regulatory variants to pig phenotypes is poorly understood. Therefore, our aim was to elucidate the molecular mechanisms by which non-coding variants cause phenotypic differences in pigs by combining evolutionary biology analyses and functional genomics.

**Results:**

We obtained a high-resolution phased chromosome-scale reference genome with a contig N50 of 18.03 Mb for the Luchuan pig breed (a representative eastern breed) and profiled potential selective sweeps in eastern and western pigs by resequencing the genomes of 234 pigs. Multi-tissue transcriptome and chromatin accessibility analyses of these regions suggest that tissue-specific selection pressure is mediated by promoters and distal cis-regulatory elements. Promoter variants that are associated with increased expression of the *lysozyme* (*LYZ*) gene in the small intestine might enhance the immunity of the gastrointestinal tract and roughage tolerance in pigs. In skeletal muscle, an enhancer-modulating single-nucleotide polymorphism that is associated with up-regulation of the expression of the *troponin C1, slow skeletal and cardiac type* (*TNNC1*) gene might increase the proportion of slow muscle fibers and affect meat quality.

**Conclusions:**

Our work sheds light on the molecular mechanisms by which non-coding variants shape phenotypic differences in pigs and provides valuable resources and novel perspectives to dissect the role of gene regulatory evolution in animal domestication and breeding.

**Supplementary Information:**

The online version contains supplementary material available at 10.1186/s12711-022-00754-2.

## Background

Throughout domestication, inbreeding and intensive artificial selection have resulted in considerable genetic and phenotypic diversity among breeds of domestic animals that offer ideal models to understand the influence of genotypic variations on phenotypes. Therefore, research on domesticated animals is crucial to improve economic traits and provide unique insights into the genetic basis of complex traits shared by humans and animals [1]. Comparative genomics and population-resequencing analyses have been performed to identify regions and genes under selection in domestic species [2–11]. However, many of the detected selective sweeps either do not contain functional coding DNA variants or are located outside the coding regions, which suggests that non-coding regulatory DNA variants play prominent roles in driving phenotypic diversity [12–16]. While mutations in the coding regions of a gene exert pleiotropic effects on many tissues, mutations in cis-regulatory elements may affect gene expression in a spatio-temporal manner [17]. Accordingly, these regulatory variants are permissible and possibly contribute to morphological evolution and phenotypic differentiation. Nevertheless, functional annotation of the non-coding genome in large animals, particularly livestock, is lacking, which limits our efforts to identify causal non-coding DNA variants that cause phenotypic differences [18].

The pig (*Sus scrofa*) is an important livestock species for food supply and an ideal model for biomedical research. Geographical divergence, local adaptation, and artificial selection have resulted in large phenotypic differences between eastern (Asia) and western (Europe and America) pigs [19–21]. Compared with western pigs, eastern pigs generally have a slower growth rate, higher fat content, better meat quality, earlier maturity, higher fecundity, greater adaptability to high roughage diets, and stronger resistance to disease [20, 21]. However, the contributions of cis-regulatory variants to phenotypic differences in pigs are poorly understood. In the present study, our aim was to elucidate the molecular mechanisms that are responsible for the large phenotypic differences between pig breeds by using a strategy based on integrative evolutionary biology and functional genomics.

First, we applied long-read sequencing (Pacbio), short paired-end reads (Illumina), Hi-C, and optical map (BioNano) technologies to generate a highly contiguous, chromosome-scale phased assembly of the genome of the Luchuan breed, which is a representative eastern pig breed. Following practices that substantially improve genome assemblies for humans, goats, and gorillas [22–24], this approach provides the first phased genome assembly with chromosome-scale contiguity for pigs. Furthermore, we present the first multi-tissue landscape of cis-regulatory genetic variations that underlie phenotypic differentiation and provide insights on the genetic background of several important traits of pigs. The novel Luchuan genome assembly, population resequencing data, and multi-tissue expression and chromatin accessibility profiling data are valuable resources to facilitate the study of pig domestication and breeding.

## Methods

### Sample collection

A male Luchuan boar was obtained from the Institute of Animal Science of Guangxi province, China, for genome assembly. Genomic DNA was extracted from a blood sample. To improve genome annotation, RNA from 14 tissues (heart, lung, subcutaneous adipose, kidney, liver, cerebrum, spleen, stomach, biceps muscle of the thigh, *longissimus dorsi* muscle, testis, ovary, large intestine, small intestine) at four developmental stages (days 0, 14, 50 and adult pigs) from four Luchuan individuals were equally pooled together. However, due to the technical difficulty of collecting some samples, samples were not available for the four developmental time-points for all tissues. The total number of tissue by time-point combinations was 37.

To analyze differentially-expressed genes between the Luchuan and Duroc breeds, ten tissues were collected from adult Luchuan and Duroc pigs (90–120 kg body weight) for RNA-seq, including skeletal muscle (*longissimus dorsi* muscle), subcutaneous adipose, cerebellum, cerebrum, heart, liver, lung, pancreas, small intestine, and stomach. Five of these tissues (cerebellum, cerebrum, skeletal muscle, small intestine, liver) were subjected to ATAC-seq experiments. Three individuals from each breed were sampled as biological replicates for RNA-seq and two for ATAC-seq, except for pancreas tissue. Tissue samples were manually dissected and then rapidly frozen in liquid nitrogen. The Luchuan and Duroc pigs were raised in the same environment on our pig farm and were sacrificed at a commercial slaughterhouse. All animal procedures were performed according to protocols approved by the Biological Studies Animal Care and Use Committee in Guangdong Province, China, and guidelines for the Care and Use of Experimental Animals established by the Ministry of Agriculture and Rural Affairs of China.

### Preparation of next-generation sequencing libraries for genome assembly

In order to generate a chromosome-scale assembly, four genome libraries were constructed and sequenced according to the manufacturers' instructions: (i) whole-genome sequencing (WGS) on the PacBio Sequel platform (20-kb library with SMARTer PCR cDNA Synthesis kit); (ii) Hi-C chromosome conformation captured reads sequencing by Phase Genomics [25] (Hi-C library were prepared with Phase Genomics Proximo Hi-C kit (Animal)); (iii) short reads paired-end sequencing (150-bp paired-end library construction with the Next Ultra DNA library prep kit) by the Illumina NovaSeq 6000 platform; (iv) BioNano optical map data (Nt.BspQI, Nb.BssSI, and DLE-1 enzymes, the library was prepared with the Bionano Prep DLS Labeling kit).

To annotate the Luchuan transcripts, two strand-specific RNA-seq libraries with an insert size of 350 bp were prepared using the NEBNext® UltraTMDirectional RNA Library Prep Kit for Illumina® (NEB, USA) and sequenced on an Illumina NovaSeq 6000 platform, to generate 150-bp paired-end reads (Berry Genomics Co., Ltd., Tianjin, China). A PacBio full-length cDNA library was constructed following the manual of the DNA Template Prep kit (Pacific Biosciences, USA) and sequenced on the Pacific Bioscience RS II sequencer (BerryGenomics, Co., Ltd., Beijing, China).

### Genome assembly with Pacbio data and Hi-C data

The primary contigs were assembled with the Falcon software packages (v2.0.5) followed by FALCON-Unzip and Arrow (v2.2.2) polishing, and then a Hi-C-based contig phasing was processed by FALCON-Phase to create phased, diploid contigs. The Phase Genomics' Proximo Hi-C genome scaffolding platform was used to create chromosome-scale scaffolds from the draft assembly using a method similar to that described by Bickhart et al. [24]. Following diploid chromosomal scaffolding using the Proximo pipeline, scaffolds were examined with the Juicebox tool (v1.8.8) [26] to correct small errors in chromosome assignment or contig ordering and orientation to improve scaffold quality. After a draft set of scaffolds was generated, FALCON-Phase was run again for Hi-C-based scaffold phasing. Finally, the Pilon (v1.22) software was used to correct errors introduced into the assembly as a result of errors in the long reads [27]. The commands that we used are listed in Additional file 1.

### Correction, ordering, and orientation of the initial assemblies by Bionano

High-molecular-weight DNA was extracted from blood samples using the Bionano Prep Blood DNA Isolation Protocol and digested with Nt.BspQI, Nb.BssSInickase, and DLE-1, respectively. After labeling and staining, DNA was loaded onto the Saphyr chip for sequencing. For each of the three enzyme libraries, respectively 453 Gb, 345 Gb, and 618 Gb of data were collected and converted into a BNX file by the AutoDetect software to obtain basic labeling and DNA length information. The filtered raw DNA molecules in BNX format were aligned, clustered, and assembled into the BNG map using the Bionano Solve pipeline. Two-enzyme (Nt.BspQI and Nb.BssSI) hybrid scaffolding was first processed to produce initial hybrid scaffolds, followed by the second round of hybrid scaffolding with a genome map of the DEL-1 enzyme. Finally, given that a relatively good reference of the Y chromosome was available for the Duroc breed (Sscrofa11.1), a reference-assisted scaffolding strategy was used to obtain chromosome-level pseudomolecules of the Y chromosome with the Chromosomer software (v0.1.4a) [28]. Quality control of the integrity of the assembly was performed by using the independent BUSCO v3 benchmark [29, 30], which specifically assesses the integrity of genic regions.

### Detection of presence–absence variations between the Luchuan and Duroc genomes and of repeats

Presence-absence variations (PAV) between the genomes of Luchuan and Duroc pigs were detected by scanPAV with default parameters [31, 32]. Short PAV ($\le 1\mathrm{kb}$) were filtered as recommended by scanPAV. Tandem repetitive sequences were identified using the Tandem Repeats Finder (version 4.07) software. The interspersed repeat contents of the Luchuan pig genome were identified using de novo repeat identification and known repeat searching against existing databases. RepeatModeler (version 1.0.8) was used to predict repeat sequences in the genome. RepeatMasker (version 4.0.7) was then used to search the Luchuan pig genome against the de novo transposable element (TE) library [33]. The homology-based approach involved the use of commonly used databases of known repetitive sequences, and the RepeatMasker (version 4.0.7) software and Repbase database (version 21) were used to identify TE in the assembled genome.

### Gene prediction and annotation

Identification of protein-coding regions and gene prediction was conducted by a combination of homology-based prediction, de novo prediction, and transcriptome-based prediction methods (for details [see Additional file 1]). Functional annotation of protein-coding genes was achieved using BLASTP (E-value 1e−05) against two integrated protein sequence databases: SwissProt and TrEMBL. Protein domains were annotated by using InterProScan (V5.30). The Gene Ontology (GO) terms for each gene were extracted with InterProScan. Pathways in which the genes might be involved were assigned by BLAST against the KEGG databases (release 59.3), with an E-value cutoff of 1e−05.

### Prediction of non-coding RNA annotations based on the Luchuan genome assembly

The tRNA genes were predicted by tRNAscan-SE (version 1.3.1), while rRNA fragments were predicted by alignment to human template rRNA sequences using BlastN (version 2.2.26) at an E-value of 1e-5. The miRNA and snRNA genes were identified by searching against the Rfam database (release 12.0) using INFERNAL (version 1.1.1). Long non-coding RNAs (LncRNAs) and circular RNAs were predicted by methods described previously [20, 34, 35].

### Identification of gene families

Orthologous gene sets in the Luchuan and Duroc pig, cattle, goat, dog, mouse, and human genomes were used for genome comparisons. BLASTP was applied to all protein sequences using a database containing protein data for these seven genomes, and the TreeFam methodology [36] was used to define a gene family and resulted in single-copy orthologous gene families for these mammalian species.

### Construction of a phylogenetic tree and estimation of divergence time among mammalian species

The single-copy gene families described above were used to construct a phylogenetic tree for the Luchuan pig and the other mammalian genomes. Four-fold degenerate sites were extracted from each family and concatenated to form one supergene for each species. The GTR + gamma substitution model was selected, and PhyML v3.0 [37] was used to reconstruct the phylogenetic tree. The divergence time among Luchuan and Duroc pigs, cattle, goats, dogs, mice, and humans were estimated using the MCMCtree program (version 4.4), as implemented in the Phylogenetic Analysis of Maximum Likelihood package, with an independent rates clock and HKY85 nucleotide substitution model. The calibration times were derived from the TimeTree database [38]. Changes in gene family size along the phylogenetic tree were analyzed by CAFE (v2.1) [39].

### Library preparation for population-based resequencing and SNP calling

Genomic DNA from the ear tissues of 16 Luchuan pigs, 18 Tongcheng pigs, and 38 Large White pigs was subjected to WGS at a coverage of at least 35X (see Additional file 2: Table S1 and Additional file 1). In addition, we downloaded the genome sequencing data of 157 western and eastern pigs with a sequencing depth of at least 10X from NCBI (see Additional file 2: Tables S2 and S3). The total downloaded data reached 8.56 Tb with an average depth of 21.14X (genome size calculated according to 2.50 G) (see Additional file 2: Tables S2 and S3) [20, 21, 40, 41].

The high-quality paired-end reads were mapped to the Duroc (Sscrofa11.1) reference genome using BWA (v0.7.12) [42]. The GATK (Genome Analysis Toolkit, v3.7-0-gcfedb67) software was used to call single nucleotide polymorphisms (SNPs). Gene-based SNP annotation was performed according to the gene annotation file Sscrofa11.1.94 for the Duroc genome using the ANNOVAR package (v2013-06-21) [43, 44] (for details [see Additional file 1]).

### Evaluation of SNP depletion in coding sequence regions

The number of SNPs in coding sequence (CDS) regions was recorded as Osnp. To determine whether SNPs are significantly depleted in CDS regions, we performed 1000 rounds of simulation to mimic the random distribution of SNPs in CDS regions. For each round of the simulations, we sampled genomic intervals from the pig genome (susScr11) excluding repeats annotated by "RepeatMasker" to match the number and length of the set of merged CDS regions and counted the number of SNPs that overlapped with these intervals, recorded as Ssnp. The P-value for testing whether SNPs are significantly depleted in CDS regions was determined by the proportion of Ssnp being smaller than Osnp.

### Phylogenetic and population genetic analyses

To analyze the population structure, we screened a subset of bi-allelic and high-quality SNPs with a call rate ≥ 90% and a minor allele frequency (MAF) ≥ 5%. A neighbor-joining (NJ) tree was constructed using the TreeBeST (v1.92) program with 200 bootstrap replicates [45]. The tree was displayed using MEGA [46]. To infer population structure, we used the ADMIXTURE (v1.3.0) software [47], which implements a block-relaxation algorithm. We filtered SNPs by testing Hardy–Weinberg equilibrium violations (P > 10^−4^) and reconstructed the model-based clustering analysis. We also performed a principal component analysis (PCA) using the program GTAC (v1.92) [48] (for details [see Additional file 1]).

### Linkage disequilibrium analysis

To estimate and compare the pattern of linkage disequilibrium (LD) of domesticated breeds, the squared correlation coefficient (r^2^) values between any two SNPs within 300-kb intervals were computed by using the Haploview (v4.269) software [49]. We produced an LD decay plot that shows the average r^2^ values in 100-bp bins against the physical distance of pairwise bins. To get reliable results, wild boars (Chinese wild boars [CWB], Korean wild boars [KWB]) and Korean black pigs [KBP], a breed with an uncertain genetic background and that is mixed with western lineages according to the admixture results, were excluded from the LD analysis and from the subsequent selective sweep analyses.

### Analysis of selective sweeps

We used multiple methods to detect regions and genes under selection. For a genomic locus, the selection is expected to increase genetic differentiation (F_ST_) between populations and to reduce nucleotide diversity (θπ) in the population in which the selective sweep occurs. SNPs with a MAF lower than 5% were removed from this analysis. Estimates of θπ and F_ST_ of eastern and western pig populations were calculated using the VCFtools (v0.1.13) package [50] with a 50-kb sliding window and a step size of 10 kb. Windows that contained less than 10 SNPs were excluded from further analysis [51]. Windows that were both in the top 10% of F_ST_ values and in the 5% left or right tails of the empirical ratio (θπ eastern/θπ western) regions were identified as regions under selection in eastern and western pigs. In addition, to avoid missing fixed signatures of selection that have both small θ_π_ values in each population and high between-population F_ST_, windows that were both in the bottom 5% of θ_π_ in each population and in the top 10% of F_ST_ were also considered as regions under selection. We combined all these regions into a set of putative regions under selection. The figure was drawn using RectChr (Version 1.24) [52]. GO enrichment analysis of genes under selection was implemented with the GOseq R package [53]. GO terms with corrected P-values < 0.05 were considered to be significantly enriched.

### Enrichment analysis of genes under potential selection in pig QTL/GWAS regions

Phenotype-associated loci derived from the pigQTLdb (updated on April 26th, 2021) [54, 55] were partitioned into quantitative trait loci (QTL) and genome-wide association study (GWAS) signals. We collected all the genes within the full length of QTL regions and within 2-Mb genomic regions centered at the midpoints of GWAS signals [56–58], respectively. Then, we used a hypergeometric test to determine whether the genes associated with a trait in the QTL/GWAS analysis were enriched in regions under potential selection (for details [see Additional file 1]).

### mRNA, lncRNA, and alternative splicing analysis of RNA-seq

Fifty-seven strand-specific RNA-seq libraries with an insert size of 350 bp were prepared using total RNA from 10 tissues (skeletal muscle, subcutaneous adipose, cerebellum, cerebrum, heart, liver, lung, pancreas, small intestine, and stomach) of Duroc and Luchuan pigs according to the manufacturer's instructions (Illumina, SanDiego, CA). The libraries were sequenced on an Illumina HiSeq 4000 platform to generate 150-bp paired-end reads (Novogene Co., Ltd., Tianjin, China). Details of these analyses are in Additional file 1.

### Western blot

Proteins were extracted from small intestine tissue using the T-PER™ Tissue Protein Extraction Reagent (Thermo Scientific™, USA) and protease inhibitor tablet (Roche, USA). The total protein concentration was quantified using the BCA protein assay kit (Invitrogen), and a western blot was performed to check the protein level of the porcine *lysozyme* (*LYZ*) gene (for details [see Additional file 1]).

### ATAC-seq analysis

ATAC-seq libraries were prepared as previously described [59] (for details [see Additional file 1]). The final library was size-selected for products of 200–600 bp using AMPure XP beads and was subjected to pair-end sequencing (PE150) on a Novaseq6000 platform. The ATAC-seq data were processed as previously described [60, 61], with two biological replicates for each tissue (for details [see Additional file 1]). We performed an analysis of motifs by using the Fimo (v4.12.0) software [62] to detect enriched DNA binding motifs of transcription factors in the ATAC-seq peaks.

To explore the enrichment pattern of differential ATAC-seq peaks, we first converted these regions to hg38 coordinates, using the LiftOver tool [63] with the parameter "minMatch = 0.1", and then performed enrichment analysis using the Genomic Regions Enrichment of Annotations Tool (GREAT) [64, 65].

### Analysis of genomic enrichment in regions under selection

To evaluate the enrichment of differentially expressed protein-coding genes (DEG) promoters in regions under selection for the 10 tissues examined, we set promoters of all protein-coding genes as a baseline group. We excluded the repeats annotated by "RepeatMasker" since they were also excluded when identifying regions under selection. To avoid spurious enrichment by functional coding variants, we removed regions under selection that are within 50 kb of functional coding variants between Luchuan and Duroc. We counted the observed value (Obs) as the number of highly differential variants between Luchuan and Duroc under selection that overlapped with promoter regions. Then, 1000 rounds of simulations were performed to evaluate the enrichment of promoters in regions under selection. For each round, we sampled genomic intervals to match the number and length of the set of promoters and recorded the number of variants mentioned above that overlapped each set of intervals as Sim for each simulation. We calculated Rand as the average of Sim over 1000 simulations. Finally, the fold of enrichment was calculated as Obs/Rand and its significance P-value was determined as the proportion of times that Sim ≥ Obs. A similar simulation strategy was used to evaluate the enrichment of ATAC-seq peaks in regions under selection, after removing ATAC-seq peaks that overlapped with promoter regions.

### Luciferase reporter assays

To generate luciferase reporter constructs for the promoter of the *LYZ* gene, we cloned the promoters (1101 bp upstream of the transcription start site (TSS)) of the Luchuan and Duroc *LYZ* gene, respectively, into the pGL3-Basic (Promega, USA) vector between the Xho I and Sac I sites by using the Homologous Recombination kit (Qingke, China). Cells isolated from the small intestine were cultured at 37 °C with Dulbecco's modified Eagle's medium/F-12 (Thermo Fisher), 10% FBS (Gibco, USA), 1% penicillin/streptomycin (Gibco, USA), and 5% CO_2_ and grown to 75 to 80% confluence in 12-well plates; then the pGL3-Luchuan-LYZ-promoter, pGL3-Duroc-LYZ-promoter, pGL3-Duroc-LYZ-modified-promoter, and pGL3-Basic (Promega, USA) vectors were each co-transfected with the pRL-TK vector (Promega, USA). Cells were harvested after 24 h and evaluated for luciferase activity using a dual-luciferase assay system (Promega, USA). Primer sequences are in Additional file 2: Table S4.

To generate luciferase reporter constructs that contain potential enhancer elements, we first randomly chose 10 skeletal muscle DEG using the sample() function without replacement in the R program and then tested the associated ATAC-seq regions (differential ATAC-seq peaks with highly differential variants in the skeletal muscle between Luchuan and Duroc) within 1 Mb of these genes in luciferase experiments. For a wide peak that included multiple highly differential variants, we tested a short DNA fragment in the luciferase experiments by centering the variant closest to the middle of the peak. These potential enhancer elements were cloned into luciferase reporter vectors (see Additional file 2: Table S5) and tested for their enhancer activity by dual luciferase experiments in C2C12 cells (for details [see Additional file 1]).

### Phenotypic analysis of skeletal muscle

Slow versus fast fiber compositions of muscle were obtained using the myofibrillar ATPase staining method [66] and microscope counting (for details [see Additional file 1]). Approximately 300 fibers per sample, which were free from tissue disruption and freeze damage, were evaluated and classified into fiber types based on MYHC1 and MYHC2b staining. The percentage of slow fibers was estimated as the ratio between MYHC1 isoform fiber and the total number of fibers counted. Microstructural changes of the skeletal muscle fibers in Landrace and Luchuan were evaluated by electron microscopy using a Zeiss DSM 962 microscope (Inspect‐F; FEI, Hillsboro, Oregon, USA) (for details [see Additional file 1]).

### RNA interference and overexpression

The synthetic siRNAs were all obtained from Genepharma (Shanghai, China; [see Additional file 2: Table S6]). The siRNAs knockdown and overexpression of *Tnnc1* were performed in mouse C2C12 cells and the expression levels of *Ho-1* and *Ogg1* were measured. The siRNAs that target *NR2F2* and the NC-siRNA were transfected into pig small intestine cells to measure the expression levels of *LYZ* after *NR2F2* knockdown. The siRNAs that target the *Tnnc1* and *Sema3g* genes, and the NC-siRNA, pcDNA3.1-Tnnc1, pcDNA3.1-Sema3g, and pcDNA3.1-Control were transfected into C2C12 cells to detect expression levels of *Myh7*, *Myh2*, *Myh4*, and *Myh1* in knockdown and overexpression experiments. To generate a *Tnnc1* overexpression vector, the 498-bp coding sequence region of the pig *TNNC1* gene was amplified using forward and reverse primers that contained BamH I and Xho I sites, respectively. The PCR products were inserted into the pcDNA3.1 (+) vector (Invitrogen, China; [see Additional file 2: Table S4]).

### Quantitative real-time polymerase chain reaction

Total RNA was extracted from small intestine pig cells using the TRIzol reagent (Invitrogen, China), according to the manufacturer's instructions, and then reverse-transcribed to complementary DNA using the HiScript III 1st Strand cDNA Synthesis kit (Vazyme Biotech, China). qRT-PCR was carried out using the SYBR Green Master Mix (Vazyme Biotech, China) and the results were analyzed using the 2△△CT method. Primer sequences are listed in Additional file 2: Table S4.

### AAV-mediated in vivo knockdown of *Tnnc1* in mice

Seven-week-old mice were obtained from the Huafukang company (Beijing, China). AAV9 serotypes of siRNA-Tnnc1 and siRNA-NT (non-target control) were produced and injected into mouse skeletal muscle. Fourteen days after injection, the tibialis anterior muscle was collected from the mice and ATPase staining was carried out with the alkaline Staining kit (Solarbio, Beijing, China) (for details [see Additional file 1]). The ATPase activity was evaluated using the staining of the cross-sectional area of muscle fiber, with a greater depth of staining indicating a higher proportion of fast muscle fibers.

### Statistical analysis for qPCR

The SPSS v20.0 (SPSS Inc, Chicago, Illinois) software was used for statistical analysis. T-tests and analysis of variance were used to assess statistical significance. A value of P < 0.05 was considered statistically significant. All experiments were repeated three times and all estimates were expressed as mean + SEM.

### Sequence and function conservation analysis of cis-regulatory elements

The differential ATAC-seq peaks between Luchuan and Duroc were defined as pig differential cis-regulatory elements (diffCREs). The software liftOver was used to convert the diffCRE (extended by 500 bp at both ends from the middle points, susScr11) to the human genome (hg38) with the parameter "minMatch = 0.5" [63, 67]. The homologous human sequences were considered as sequence conserved diffCREs. A diffCRE from a pig tissue was considered functionally conserved if its human counterpart has DNase signals in the concordant human tissue (ENCODE v5). To investigate the characteristics of these functionally conserved sequences, we performed a GO biological process analysis using the GREAT software [64, 65]. In addition, we performed a comparison of GWAS signals by overlapping these functionally conserved homologous sequences with human GWAS signals (50 kb genomic regions centered at GWAS tag SNPs to account for LD).

## Results

### De novo assembly of the Luchuan pig genome

We generated a highly-contiguous, chromosome-scale phased assembly of the Luchuan pig by applying long-read sequencing, short paired-end read sequencing, Hi-C, and optical map technologies (see Additional file 2: Table S7 and Additional file 3: Fig. S1). The 2.58 Gb primary haplotype-specific contig assembly had a contig N50 of 18.03 Mb and a scaffold N50 of 140.09 Mb (see Additional file 2: Table S8) and its quality was comparable to that of the Duroc genome and higher than those of previously published pig genomes (Table 1) [20, 51, 68, 69]. The alternative haplotig assembly size was very close to the primary assembly size, with a contig N50 of 17.77 Mb and a scaffold N50 of 140.08 Mb. The pseudo-chromosomes of the Luchuan pig presented high collinearity with the Sscrofa11.1 reference genome, providing further evidence supporting the quality of our genome assembly (see Additional file 3: Fig. S2). By comparison, approximately 51.51 Mb of the Luchuan assembly was absent in the Sscrofa11.1 reference. The lengths of most of the sequence differences between the Luchuan and Duroc assemblies were shorter than 10 kb (see Additional file 2: Table S9). Two hundred and eighty-nine predicted protein-coding genes were located in these regions and their functions were mainly related to collagen alpha chain-like and spidroin-2-like functions (see Additional file 2: Table S10). This new genome assembly provides a valuable resource to the pig genetics community for studies on allele-specific gene expression, epigenetic regulation, genome structure, and evolution.

**Table 1:** Comparison of genomic features between whole genome assemblies of the Luchuan breed and other pigs

	Luchuan	Duroc [105]^a^	Tibetan wild [51]	Wuzhishan [69]	Bama [68]
Sequenced genome size (Gb)	2.58	2.50	2.43	2.64	2.49
Contig N50 (Mb)	18.03	41.89	0.0207	0.0235	1.01
Scaffold N50 (Mb)	140.09	138.97	1.06	5.43	140.44
Percentage of anchoring and ordering	96.1%	97.34%	–	–	97.49%
Predicted PCG	22,710	22,452	21,806	20,326	21,334
Repeat proportion (%)	40.16	40.55	39.47	38.20	37.32
Complete BUSCOs (%)	95.1	96.0	93.1	95.2	93.9

^a^Statistic of the Duroc pig genome was based on Sscrofa11.1 (Ensembl release-95)

Percentage of anchoring and ordering: the percentage of assembled final assembly was assigned to the 20 pig pseudo-chromosomes (18 autosomes and X/Y chromosome)

PCG: protein-coding genes

The numbers in brackets represent references

We constructed a phylogenetic tree for the Luchuan and Duroc pig genomes and five other mammalian genomes (cattle, goats, dogs, humans, and mice). The Luchuan assembly showed substantially fewer events of gene family expansion than the Duroc reference genome (63 vs. 433) and more events of gene family contraction (560 vs. 161) than the genome of the most recent common ancestor of the Luchuan and Duroc breeds (see Additional file 3: Fig. S3). GO enrichment analysis revealed that the expanded genes in the Luchuan pig were closely related to responses to oxidative stress and biotic stimuli, which may reflect that the Luchuan breed has been under selective pressure to adapt to free-range environments. The contracted genes in the Luchuan pig genome were significantly enriched (P < 0.05) in GO terms for olfactory receptor activity, sensory perception of taste, and blood coagulation (see Additional file 2: Table S11). This finding is consistent with a previous study that reported that the genome of the Duroc breed contains more olfactory-related genes compared to the Luchuan breed and that this breed may display a better response to a wider range of food than the Luchuan breed [51].

### Population structure and selective sweep analyses of eastern and western pigs

**Fig. 1:**
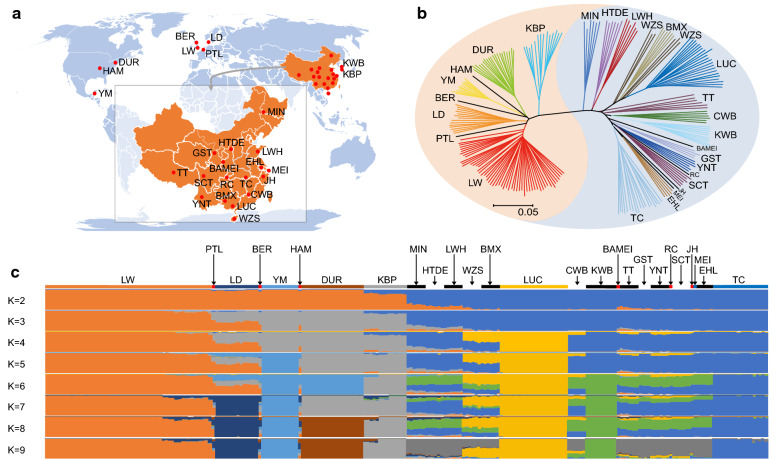
Genetic divergence of 234 eastern and western pigs. **a** Geographical distribution of the pigs used in our study. BAMEI: Bamei; BER: Berkshire; BMX: Bamaxiang; CWB: Chinese wild boar; DUR: Duroc; EHL: Erhualian; GST: Tibetan (Gansu); HAM: Hampshire; HTDE: Hetao; JH: Jinhua; KBP: Korean black pig; KWB: Korean wild boar; LD: Landrace; LUC: Luchuan; LW: Large White; LWH: Laiwu; MEI: Meishan; MIN: Min; PTL: Pietrain; RC: Rongchang; SCT: Tibetan (Sichuan); TC: Tongcheng; TT: Tibetan (Tibet); WZS: Wuzhishan; YM: Yucatan Miniature; YNT: Tibetan (Yunnan). **b** A neighbor-joining tree constructed using SNP data. **c** Genetic structure of 234 pigs. The number of ancestral populations (K) was predefined from 2 to 9

We collected 234 individuals from 26 breeds (11.99 Tb resequencing data, with an average of 20.50X coverage [ranging from 17.58X to 45.69X]) to identify putative positive signatures of selection in the genomes of domesticated pigs (Fig. 1a) and (see Additional file 2: Tables S1 to 3). The sequencing data were aligned to the Duroc reference genome and 26.64 × 10^6^ SNPs were identified across the genome. These high-quality SNPs were mostly located in intergenic (60.3%) and intronic (37.0%) regions and were rarely located in coding sequences (0.6%) (see Additional file 2: Table S12). The significant depletion of SNPs in coding regions (P < 0.001) (see Additional file 2: Table S12) indicates that these regions have been under strong purifying selection. In addition, 3.74 × 10^6^ indels were identified, of which 4238 (0.1%) were located in exonic regions and may exert a large impact on protein structure.

The population structure of the 234 pigs was characterized using NJ phylogenetic reconstruction, STRUCTURE, and PCA (Fig. 1bc and [see Additional file 3: Fig. S4 and Additional file 2: Table S13]). Based on the results of these analyses, all western and eastern pigs (except for KBP) were divided into two clades. The KBP clade was relatively closer to the western pig clade (Fig. 1b) and had more western than eastern pig lineages (K = 2, 3, and 4 in the Admixture analysis, see Fig. 1c), which may be the result of modern breeding practices. In general, our analysis of population structure provided further evidence for the independent domestication of western and eastern pigs [21, 70]. The number of SNPs and indels was larger in eastern pigs (22.87 × 10^6^ and 3.23 × 10^6^, respectively) than in western pigs (16.40 × 10^6^ and 2.52 × 10^6^, respectively).

**Fig. 2:**
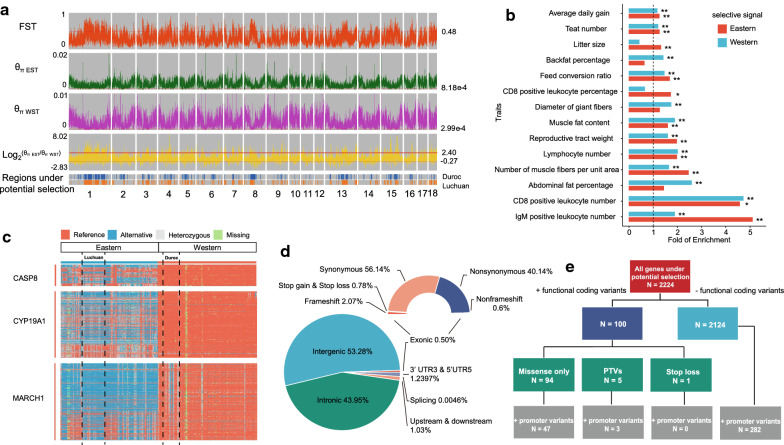
Genes under potential selection in pigs. **a** The distribution of fixation index (F_ST_), nucleotide diversity (θ_π_), and putative regions under selection (Blue for Duroc and Orange for Luchuan) on the 18 pig autosomes. **b** Enrichment pattern of putative genes under selection in QTL regions that are associated with several important economic traits. Red represents genes located in regions under selection of eastern pigs while blue represents those of western pigs. * represents FDR < 0.05. ** represents FDR < 0.01. **c** The coding haplotypes of three candidate causal genes with highly differential functional coding variants across eastern and western pigs. **d** Annotations of highly differential variants under potential selection. **e** The proportion of genes under potential selection with different types of highly differential variants

Nucleotide diversity and LD were calculated for eastern and western pigs using the 25.08 × 10^6^ autosomal SNPs. Eastern pigs had higher polymorphism levels (median [θ_π, eastern_/θ_π, western_] = 1.47) and LD decay levels (see Additional file 3: Fig. S5) than western pigs, which suggests that eastern pigs had a larger effective population size than western pigs. Potential selected regions in eastern and western pigs were identified by calculating F_ST_ and θ_π_ (Fig. 2a, see Methods for details). In total, regions covering 135.4 Mb (accounting for 5.4% of the pig genome with 1797 genes) with strong selective sweep signals were identified in western pigs, compared to 56.8 Mb (accounting for 2.3% of the pig genome with 1026 genes) in eastern pigs (see Additional file 2: Tables S14, 15). This difference suggests that natural or artificial selection has been stronger in western pigs than in eastern pigs.

Genes under potential selection are enriched in QTL regions for several important economic traits in the pig based on PigQTLdb, which are relevant to meat production, meat quality, disease resistance, and reproduction. Similar enrichment patterns were observed for the pig GWAS dataset (PigQTLdb) [54, 55] (Fig. 2b) and (see Additional file 3: Fig. S6 and Additional file 2: Table S16). These enrichment patterns provide clues for linking genetic divergence with phenotypic improvement. Genomic regions associated with QTL and selective sweeps are large and contain multiple genes. We used GWAS signals and highly differential (delta allele frequency [deltaAF] ≥ 0.9) functional coding variations to identify candidate causal genes in these regions (see Additional file 2: Table S17a). For example, immunity-related GWAS and QTL signals were found to be associated with the *CYP19A1* gene [71, 72], which is under potential selection in eastern and western pigs possibly through a highly differential missense mutation (chr1:120549953) (Fig. 2c). The *CYP19A1* gene encodes aromatase, an enzyme that functions in estrogen biosynthesis. A previous study indicated that estrogen is responsible for modulating the immune response [73]. The *MARCH1* gene is also under potential selection in eastern and western pigs, possibly through a frameshift mutation (chr8:52387385) (Fig. 2c), and has been associated with growth rate in GWAS and QTL studies [74, 75]. Knockout of *MARCH1* leads to excessive weight gain and visceral adiposity by regulating lipid metabolism and glucose tolerance [76]. The *CASP8* gene affects the risk of obesity and diabetes in mice by regulating whole-body glucose metabolism [77]. Selection of *CASP8* in western pigs might be caused by the presence of two highly differential missense mutations in this gene (chr15:104938018 and chr15:104944628) (Fig. 2c). The *CASP8* gene has been associated with back fat and muscle fiber in QTL studies but not in GWAS [78, 79].

Unlike GWAS in human populations, current pig GWAS are rare (444 GWAS were collected in the pigQTLdb) and are based on small sample sizes [75, 80, 81]. This lack of power was exacerbated when we focused on the highly differential variants between eastern and western pigs. Pig GWAS have usually been performed in a population of western or eastern pigs. Thus, variants that are nearly fixed in either population lack statistical power to be detected in GWAS. To avoid these lack-of-power issues, we focused on functional annotations and experimental follow-ups to fine-map potential causal variants or genes.

In our previous analyses, annotating coding variants facilitated the interpretation of signatures of selection. However, in regions under potential selection, 99.5% of the SNPs or indels were located outside exons, which suggests an important but little-explored role of cis-regulatory variants in driving selection (Fig. 2d). Consistently, only 4.5% of the genes under potential selection had functional coding variants that differed substantially between eastern and western pigs (Fig. 2e). The remaining genes were either in LD with nearby causal genes or were subject to selection of regulatory mutations that modulate their expression levels. Among these genes, 13.3% had highly differential promoter variants and may have been subject to non-coding selection forces that act on their promoters (Fig. 2e). Furthermore, 94 genes under selection showed highly differential missense coding and promoter variants. We speculate that some of these genes represent a successive accumulation of beneficial coding and regulatory variants during natural and artificial selection. Notably, only a few protein-truncating variants (PTV) (frameshift and stop gain) were under potential selection, which suggests that protein inactivation is not a major player in pig domestication (Fig. 2e), consistent with previous studies in the domestication of chickens and rabbits [4, 11].

### Roles of *LYZ* promoter variants in increased gastrointestinal immunity and roughage tolerance

**Fig. 3:**
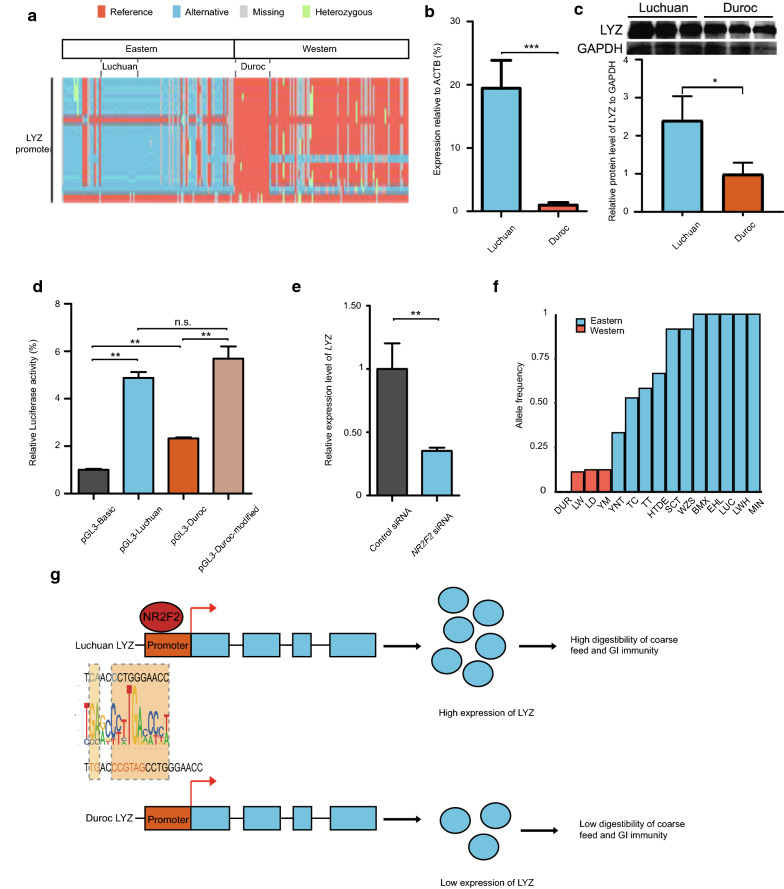
Differential promoter variants of the *LYZ* gene might contribute to high roughage digestibility and gastro-intestinal immunity in Luchuan. **a** Haplotypes of *LYZ* promoter variants across all eastern and western pigs. **b** Comparing the expression level of *LYZ* between the Luchuan and Duroc breeds by qPCR. **c** Comparing the protein level of LYZ between Luchuan and Duroc. **d** Luciferase reporter assays comparing the promoter activity of promoter sequences from Luchuan (pGL3-Luchuan), from Duroc (pGL3-Duroc), and from Duroc modified with the Luchuan promoter allele (one indel and two adjacent SNPs) (pGL3-Duroc-modified). **e** Comparing the expression level of *LYZ* before and after knockdown of *NR2F2.* **f** Frequencies of the Luchuan promoter allele (two adjacent SNPs and one indel) across different pig breeds. DUR: Duroc pig; LW: Large White pig; LD: Landrace pig; YM: Yucatan miniature pig; YNT: Tibetan pig (Yunnan); TC: Tongcheng pig; TT: Tibetan pig (Qinghai-Tibetan); HTDE: Hetaodaer pig; SCT: Tibetan pig (Sichuan); WZS: Wuzhishan pig; BMX: Bamaxiang pig; EHL: Erhualian pig; LUC: Luchuan pig; LWH: Laiwu black pig; MIN: Min pig (Shandong). **g** A model of how the genetic differentiation of the *LYZ* promoter might contribute to the adaption of Luchuan to a less clean environment and plant-enriched roughage feeding. The sequence divergences between Luchuan and Duroc are shaded in light yellow

Interpreting selection on non-coding variants is challenging because of insufficient functional annotations. To understand the mechanisms by which non-coding variants cause phenotypic differentiation between eastern and western pigs, we explored the biological functions of such variants in the Luchuan and Duroc breeds. To ensure the reliability of regions under selection, we filtered for highly differential alleles between these two breeds (DeltaAF ≥ 0.9) and prioritized genes with GWAS signals relevant to pig economic traits (see Additional file 2: Table S17b). Exploration of the genes in these refined regions under selection revealed the *lysozyme* (*LYZ*) gene, with 28 highly differential DNA variants in its promoter region (chr5:33611592–33613593, Fig. 3a, and [see Additional file 2: Table S18]). Lysozyme breaks the bonds in the peptidoglycan layer of the outer membrane of bacterial cell walls. It protects cells against bacterial pathogens and regulates interactions between the gut microbiota and host immune systems [82, 83]. In the digestive system, lysozyme releases plant fermentation-derived nutrients from within the bacteria [84, 85]. Genetic differentiation analysis revealed different haplotypes in *LYZ* between the Luchuan and Duroc breeds (Fig. 3a). The *LYZ* gene (θ_π, Luchuan_ = 1.65E−04) was at the bottom 5% of the θ_π_ regions in Luchuan pigs. In the small intestine, the mRNA and protein expression levels of *LYZ* were significantly higher in Luchuan than Duroc pigs (Fig. 3bc). Luciferase reporter assays revealed that the Luchuan *LYZ* promoter up-regulates the expression of the reporter gene in pig small intestine cells (Fig. 3d). The high activity of the *LYZ* promoter mainly resulted from one indel (chr5:33612231–33612236) and two adjacent SNPs (chr5:33612227 and chr5:33,612,228) that were differentially fixed in Luchuan and Duroc pigs. These variants created a transcription factor binding motif cognate to NR2F2 (motif score 9.9, q-value 0.00249), a member of the steroid thyroid hormone superfamily of nuclear receptors. The NR2F2 protein regulates certain intestinal-specific genes by binding to their promoters [86]. Knockdown of *NR2F2* significantly decreased the expression of *LYZ* in pig small intestine cells (Fig. 3e). Furthermore, this Luchuan allele showed a higher frequency in many other eastern breeds compared to western breeds (Fig. 3f). Therefore, selection of the genetic variants that result in higher *LYZ* promoter activity might enhance intestinal anti-microbial activities and the capacity of the Luchuan and many other eastern pig breeds to digest coarse feed (Fig. 3g).

### Skeletal muscle cis-regulatory elements enriched for variants under potential selection

**Fig. 4:**
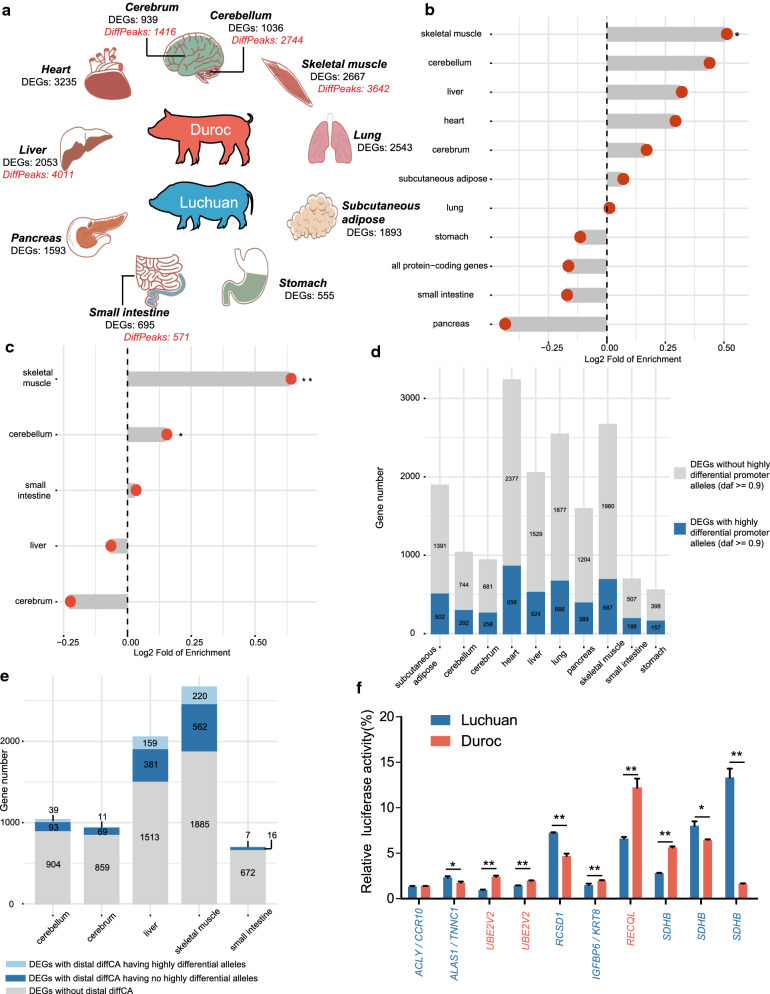
Integration of ATAC-seq and RNA-seq data to understand how cis-regulatory DNA variants might underlie pig phenotypic differentiation. **a** Experimental design and sample collection for the RNA-seq and ATAC-seq analysis in Luchuan and Duroc pigs. **b** Enrichment of DNA variants under potential selection in DEG promoters. The enrichment of promoters of all protein-coding genes was set as a baseline group. * Represents a p-value < 0.05. **c** Enrichment of DNA variants under potential selection in ATAC-seq peaks with promoter regions excluded. * represents a p-value < 0.05. ** Represents a p-value < 0.01. **d** Number of DEG with highly differential promoter variants. **e** Number of DEG with distal diffCA having highly differential variants. **f** Luciferase reporter assays in C2C12 cells to evaluate the effect of highly differential DNA variants on enhancer activity. The genes on the x-axis labeled in blue are more highly expressed in the skeletal muscle of Luchuan while those in orange are more highly expressed in Duroc. ** represents a p-value < 0.01, * represents a p-value < 0.05

Genetic differentiation and potential selection of *LYZ* promoter variants led us to further study the mechanisms by which non-coding genetic variants influence phenotypic diversity. We performed RNA-seq analyses in 10 tissues (skeletal muscle, subcutaneous adipose, cerebellum, cerebrum, heart, liver, lung, pancreas, small intestine, and stomach) of Luchuan and Duroc pigs and identified DEG and lncRNAs between the two breeds (Fig. 4a) and (see Additional file 2: Tables S19–22). The functions of DEG in each tissue were closely associated with the phenotypes of the two breeds (see Additional file 3: Fig. S7). For example, in muscle, 1401 and 1266 genes were, respectively, up- and down-regulated in Luchuan pigs compared to Duroc pigs (see Additional file 3: Fig. S8a). The up-regulated genes were significantly enriched in muscle contraction functions (see Additional file 3: Fig. S8b), while the down-regulated genes were significantly enriched in cell cycle functions (see Additional file 3: Fig. S8c). This differential expression of genes could be the result of the differential promoter activity that is caused by differential promoter variants between Luchuan and Duroc pigs. After accounting for highly differential functional coding variants (see Additional file 1), DEG promoters in the skeletal muscle were most significantly enriched in the highly differential variants that were under potential selection (Fig. 4b) and (see Additional file 2: Table S23). The muscle DEG with highly differential promoter alleles included the *VDR*, *ACTC1*, and *SHOC2* genes, which have been associated with intramuscular fat content, residual feed intake, and weight gain, respectively [87–92]. Similar to the *LYZ* promoter alleles, these promoter alleles were highly differentially distributed in other eastern and western pig breeds (see Additional file 3: Fig. S9]. In the other tissues, we also identified differentially expressed genes that did not have highly differential functional coding but promoter variants under potential selection. These genes represent potential selection targets of gene-proximal promoter variants (see Additional file 2: Table S24).

To understand the potential selection of distal cis-regulatory elements, we performed ATAC-seq experiments in five tissues (skeletal muscle, cerebellum, cerebrum, liver, and small intestine) of Luchuan and Duroc pigs (Fig. 4a). Results showed open chromatin regions with signal strengths that depended on enhancer activity (see Additional file 2: Tables S25–27 and Additional file 3: Fig. S10]. After accounting for highly differential functional coding variants and excluding promoters, the open chromatin regions in the skeletal muscle, which represent putative distal cis-regulatory elements, were most significantly enriched for highly differential variants in potential regions under selection (Fig. 4c) and (see Additional file 2: Table S28). One possible explanation is that economic traits in the pig that are relevant to skeletal muscle development and growth, such as meat production and quality, were subject to the strongest selection, targeting cis-regulatory elements in a tissue-specific manner. Differential ATAC-seq peaks between skeletal muscle of Luchuan and Duroc pigs were significantly enriched in muscle fiber development, which is highly relevant for economic traits in the pig (see Additional file 2: Table S29). The enrichment of highly differential variants in differential ATAC-seq peaks [log_2_(fold of enrichment) = 0.93, P < 0.001] was even higher than in all ATAC-seq peaks in the skeletal muscle [log_2_(fold of enrichment) = 0.64, P < 0.001] (see Additional file 2: Table S28). Moreover, promoters associated with DEG and differential ATAC-seq peaks showed the highest enrichment signal in the skeletal muscle (Fig. 4b and 4c) and (see Additional file 2: Tables S23 and S28).

In the differential open chromatin regions, highly differential SNPs or indels in Luchuan and Duroc pigs were likely causal cis-regulatory DNA variants that drive differential gene expression. To identify such causal variants, we screened for differential open chromatin regions that overlapped with selected regions within 1 Mb of DEG in each tissue (see Additional file 2: Table S30). Although DEG for the stomach tissue showed the highest proportion (28.3%) of highly differential promoter alleles (Fig. 4d), skeletal muscle DEG revealed the highest proportion (8.25%) of highly differential SNPs or indels with distal differential chromatin accessibility (diffCA; Fig. 4e). Thus, our analyses further suggest that selection for meat-relevant traits may more extensively target distal cis-regulatory variants in skeletal muscle than in the other tissues. From the 808 skeletal muscle distal diffCA regions with highly differential DNA variants (see Additional file 2: Table S30), we randomly selected 10 to test for enhancer activity using luciferase reporter assays in C2C12 cells, of which nine showed significant differences in luciferase activity. Seven of these nine regions displayed an enhancer activity, as well as an ATAC-seq signal, and the level of expression of their potential target genes differed in the same direction (Fig. 4f) and (see Additional file 2: Table S5).

### Contribution of the *TNNC1*-enhancer variant to differences in meat quality

**Fig. 5:**
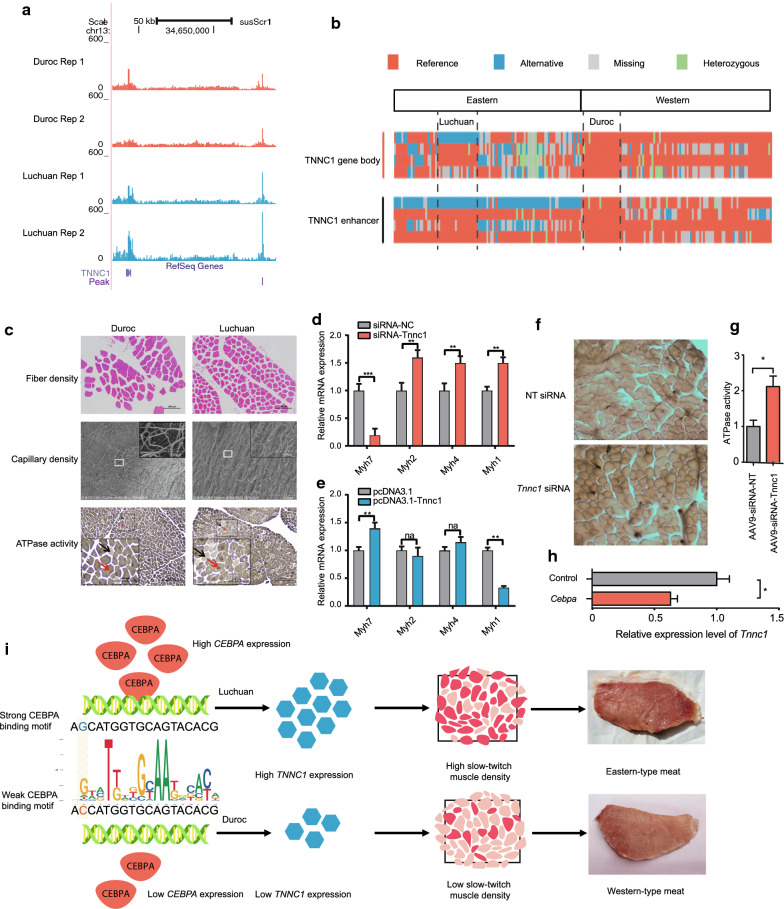
Differential regulation of *TNNC1* expression may result in differences in meat quality between eastern and western pigs. **a** Genome browser view of the differential ATAC-seq peak near the *TNNC1* promoter. **b** Haplotypes of the *TNNC1* gene body and enhancer across all eastern and western pigs. **c** Characteristics of the skeletal muscle in Luchuan and Duroc pigs. In the sub-panel of fiber density, structural differences in skeletal muscle are revealed by the environmental scanning electron microscope. In the sub-panel of capillary density, scanning electron microscope images of the muscle fiber bundle in Luchuan and Duroc pigs show the orientation and tortuosity of branching vessels at different hierarchies. In the sub-panel of ATPase staining, black arrows exemplify the slow muscle fibers which are lightly stained due to lower ATPase activity, while red arrows exemplify the fast muscle fibers which are heavily stained due to higher ATPase activity, magnification of ×40. **d** Expression of slow muscle (*Myh7*) and fast muscle (*Myh2, Myh4*, and *Myh1*) markers after siRNA knockdown of *Tnnc1* in C2C12 cells. **e** Measuring the same markers as **d** after overexpression of *Tnnc1* in C2C12 cells. **f** ATPase staining of mice skeletal muscle two weeks after in vivo injection of AAV-mediated non-target control (NT siRNA) and *Tnnc1* siRNA, respectively*.* **g** Barplots comparing the intensity of ATPase staining for samples under the same treatment as in **f**. **h** Comparing the expression level of *Tnnc1* before and after *Cebpa* knockdown in C2C12 cells. **i** A model of potential selection on a regulatory mutation in one enhancer of the *TNNC1* gene in the Luchuan pig. The Luchuan allele generates a stronger CEBPA transcription factor binding motif, which increases the expression of *TNNC1*, resulting in a higher proportion of slow-twitch muscle and meat quality improvement, including better color appearance, higher water-holding capacity, tenderness, and marbling (See Discussion for more details). The regulatory allele might be further favored during selection by a higher expression of *CEBPA* in the Luchuan breed. The sequence divergence between the Luchuan and Duroc breeds is shaded in light yellow

One prominent example of a relationship between cis-regulatory variations and phenotypic differentiation is the *TNNC1* gene, which displays a potential putative signature of selection among the bottom 5% of θ_π_ genome regions (θ_π,Luchuan_ = 1.65E−04) and for which a GWAS signal relevant to total fat content and back fat has been detected in pig [54, 55, 93, 94]. Further evidence in favor of its potential role in meat quality was provided by functional analysis. TNNC1 is a central regulatory protein of striated muscle contraction and a core component of slow-twitch muscle fiber [95–97]. The expression of *TNNC1* was higher in the muscle tissue of Luchuan than of Duroc pigs (see Additional file 2: Table S20). Only one putative cis-regulatory region was detected that is under potential selection in both eastern and western pigs, ~ 87.6 kb from the TSS of *TNNC1*. The chromatin accessibility of this region was greater in the skeletal muscle of Luchuan than of Duroc pigs (see Fig. 5a, false discovery rate (FDR) of 0.012). Interestingly, in this region there was only one SNP (chr13:34681950) with highly differentiated alleles between eastern and western pigs and it had alternate fixed alleles in Luchuan and Duroc pigs (Fig. 5b). The Luchuan allele of this SNP increased the enhancer activity by ~ 25% (Fig. 4f). Compared with Duroc pigs, Luchuan pigs had a higher proportion of slow-twitch muscles, denser capillary vessels, and stronger oxidative capacity (Fig. 5c) and (see Additional file 3: Fig. S11). Manipulating the expression of *Tnnc1* in vitro and in vivo suggested that troponin C1 regulated the transition of slow/fast muscle fibers (Fig. 5d–g) and (see Additional file 3: Fig. S12]. The stronger enhancer activity of the Luchuan allele compared to the Duroc allele results in a stronger CEBPA transcription factor binding motif (motif scores were 6.0 [q-value 0.0008] and 2.9 [q-value 0.002] for Luchuan and Duroc pigs, respectively). In mouse muscle cells, induction of slow fibers by overexpression of *Myh3* induced the expression of *Cebpa* [98]. In our study, the expression of *Tnnc1* decreased after in vitro knockdown of *Cebpa* in C2C12 cells (Fig. 5h)*.* Thus, CEBPA might act upstream of *TNNC1* and the potential selection of the regulatory allele that favors CEBPA binding in Luchuan pigs is expected to up-regulate *TNNC1* expression, which consequently increases the proportion of slow muscle fibers. *CEBPA* expression was higher in Luchuan than in Duroc pigs (see Additional file 2: Table S20), which might confer a further selective advantage to the Luchuan regulatory allele by reinforcing its transcriptional stimulatory signal to further boost the expression of *TNNC1*. Several meat quality traits, including red color appearance, high water-holding capacity, tenderness, and marbling are associated with a high proportion of slow muscle fibers [99–101]. Furthermore, reactive oxygen species metabolism has been found to be associated with the meat quality of pork, beef, and broilers [102–104] and we found that *Tnnc1* negatively regulates the expression of oxidative stress markers (*Ho-1* and *Ogg1*) in vitro (see Additional file 3: Fig. S13).

Although *TNNC1* was the nearest DEG to the regulatory variant that alters the CEBPA-binding motif (chr13:34681950), another nearby gene, *SEMA3G* (96.3 kb away), was up-regulated in Luchuan pigs (see Additional file 2: Table S20). Similar to *Tnnc1*, manipulating the expression of *Sema3g* in C2C12 cells affected the expression of several fast/slow muscle markers (see Additional file 3: Fig. S14], indicating that *SEMA3G* mediates the muscle-fiber transition effect of the regulatory variant (chr13:34681950). However, expression of *Sema3g* was not affected by in vitro knockdown of *Cebpa* (see Additional file 3: Fig. S14). In addition, we observed the presence of an intronic SNP with significantly different alleles (chr13:34591944) between Luchuan and Duroc pigs (Fig. 5b). However, this SNP did not exhibit diffCA and *TNNC1* showed no differential isoform expression in the skeletal muscle between the two breeds (see Additional file 2: Table S22). Thus, this intronic SNP is not likely to have been under selection or to be involved in meat quality. Taken together, our results suggest that differences in meat quality between eastern and western pigs can be partly explained by differential selection on a regulatory SNP that affects muscle fiber composition in *cis* and this selection might be further favored by up-regulating an upstream transcription factor in *trans* (Fig. 5i).

## Discussion

Eastern and western pigs have been subject to separate domestication processes and exhibit phenotypic and genomic differences [20, 69, 70]. Such differences provide abundant resources to understand the influence of DNA variants on phenotypes and offer tremendous opportunities to identify the genetic basis of complex traits and diseases in the pig. In the current study, we assembled a high-quality genome of the Chinese indigenous Luchuan pig and identified 26.64 × 10^6^ SNPs and 3.74 × 10^6^ indels from whole-genome resequencing data of 234 pigs, including eastern and western pigs. Genetic differentiation between the Luchuan and Duroc pigs provides a useful resource to understand the genetic basis that underlies phenotypic differentiation in pigs. By generating and exploiting multi-tissue gene expression and chromatin accessibility data, we highlighted the importance of non-coding variants in shaping phenotypic differences in pigs and proposed candidate genes and regulatory sequences that might have been subject to selection. Extending our strategy to other livestock species with a lower inter-subspecies genetic differentiation than pigs might weaken its strength. However, this strategy is still more effective than using only resequencing data for fine mapping putative regulatory variants and causal genes. In our study, combining multi-omics information with data on population-specific variants led us to provide an effective interpretation of the genetic and phenotype variations in pigs and other livestock. It also opened a novel perspective to dissect the molecular mechanisms that underlie economic traits in livestock.

The current pig reference genome (Sscrofa11.1) was derived from a western Duroc pig [105]. Compared with western pigs, eastern pigs have different genetic backgrounds and thus require their own reference genomes for an improved characterization of their genetic variation. However, the quality of the current genome assemblies for Chinese indigenous pigs is not comparable to that for Duroc pigs [20, 51, 68]. Recently, the rapid progress in long-read sequencing, Hi-C, and assembly methods has enabled the generation of phased genome assemblies with chromosome-level quality [106–108]. Building on these technological breakthroughs, we present the first phased chromosome-scale genome assembly of pigs. Our genome assembly yielded a 2.58 Gb primary assembly with a contig N50 of 18.03 Mb, and its quality was comparable to that of the current reference genome [105] and even superior to that of a recently published chromosome-level genome of Chinese indigenous Bama pigs [68].

As techniques for genome assembly and whole-genome resequencing become increasingly accessible, identification of highly differential genomic regions has become routine as the initial step in understanding the genetic basis of domestication. However, these regions usually contain multiple DNA variants, most of which are hitchhikers without biological effects. In addition, only a few functional coding variants might explain the signals of potential selection [12–16]. These challenges have rendered the fine mapping of the underlying causal variants and genes extremely difficult. Recently, a few studies have addressed these challenges by using annotated putative cis-regulatory sequences, with the rationale that positive selection might act on non-coding sequences that regulate the expression of key genes. However, these studies used either conserved non-coding regulatory DNA elements that lack tissue-level resolution [16, 109] or epigenomic markers associated with regulatory activity in one or two tissues [14]. In the current study, we profiled the gene expression and chromatin accessibility landscape across multiple pig tissues. By harnessing this abundant set of functional annotations, our approach demonstrated unprecedented power in elucidating the genetic basis of pig domestication and breeding. To the best of our knowledge, this study is the first to use multi-tissue functional genome annotations to explore the contribution of non-coding variants to phenotypic variation in livestock. Furthermore, due to the conservation of important biological processes between mammals, cis-regulatory elements in pigs might shed light on the genetic basis of human traits. For instance, 91.0% of the differential open chromatin regions between Luchuan and Duroc pigs had conserved sequences in the human genome, of which 61.6% were functionally conserved. These functionally conserved human sequences were significantly enriched in muscle-related biological processes, including "regulation of skeletal muscle adaptation" and "regulation of glycolytic process". In addition, 70.7% of these functionally conserved homologous sequences overlapped with human GWAS signals (50 kb genomic regions centered at GWAS tag SNPs to account for LD). Some of these overlapped with traits relevant to muscle, including "Obesity-related traits", "Body mass index", and "Body fat percentage".

In Luchuan pigs, we found a set of promoter variants that increased activity of the *LYZ* promoter and that were associated with increased *LYZ* expression. The *LYZ* gene encodes the lysozyme that is present in the small intestine of pigs. In piglets, dietary supplements of lysozyme improve development and function of the intestine, suppress inflammatory responses, and enrich beneficial bacterial composition [83, 110–112]. Adaptive evolution of lysozymes has facilitated the digestion of plant-based materials in ruminants and Colobinae monkeys [113, 114]. Before the advent of the modern breeding industry in China, pigs were often provided with unhygienic environments and plant-enriched roughage feeding. Therefore, selecting for high *LYZ* expression in the Luchuan and many other eastern indigenous pigs might have helped them adapt to poor living conditions. Comparison of different eastern breeds with different Luchuan *LYZ* allele frequencies would further strengthen our conclusion. However, publicly available data on the performance of disease resistance and roughage tolerance in different eastern breeds are insufficient. Furthermore, different eastern breeds are raised in different geographic locations with different forage sources, and standardized protocols to measure the relevant phenotypes are still lacking. These factors severely affect the comparison between different eastern breeds. Enrichment of variants under potential selection in the skeletal muscle was observed in the promoter regions and in distal cis-regulatory elements predicted by promoter-excluded ATAC-seq peaks. This enrichment pattern suggests that modulating cis-regulatory activity may confer tissue specificity during trait selection. We proposed a model in which a non-coding SNP allele that drives the differential enhancer activity of *TNNC1* might have been differentially selected during breed development in eastern and western pigs. Selection in Luchuan pigs might have been further enhanced by up-regulating the expression of *CEBPA*, an upstream regulator of *TNNC1*. A high proportion of slow fibers is associated with red color, high water-holding capacity, tenderness, and marbling (high intramuscular fat) [99–101]. This stands in contrast to the selection for lean muscle and high growth rates in western commercial pigs, resulting in selection for a higher proportion of fast than slow fibers [115, 116].

Apart from protein-coding genes, we also identified a set of lncRNAs under selection that exhibit differential expression, splicing, or chromatin accessibility in Luchuan and Duroc pigs (see Additional file 2: Table S31). For example, the level of expression of XLOC_000472, which is a lncRNA showing increased expression and chromatin accessibility in the liver of Luchuan pigs, was positively correlated with that of its neighbor, the hepatic *lipase* gene (R = 0.49). XLOC_000472 was also differentially spliced between Duroc and Luchuan pigs in the liver (see Additional file 2: Table S31). The highly differential non-coding SNPs of XLOC_000472 might be causal cis-regulatory variants that affect expression or splicing. These results provide experimental evidence supporting the role of cis-regulatory variations in driving phenotypic differentiation during pig domestication and improvement. We found several differentially spliced genes with differential SNPs within 2 bp of the corresponding splicing junctions (see Additional file 2: Table S32). This finding is consistent with previous studies suggesting the limited role of isoform alteration in the domestication of livestock animals [4].

In our previous study, we found that chromatin accessibility and naked-sequence-dependent reporter-driving activity co-determine the endogenous activity of a potential enhancer [117]. Thus, the eight regions with consensus ATAC-seq and luciferase differences indicated that the relevant variants have the expected differential endogenous regulatory activity (see Additional file 2: Table S5). Interpretation of the two remaining peaks is challenging. The decoupling of chromatin accessibility and naked-sequence-dependent enhancer activity might result from the fact that the cellular context of the luciferase reporter system still differs to some extent from that of the pig, which is a major limitation of most functional genomics validation studies.

In addition to chromatin accessibility and gene expression, our analysis framework readily extends to the adoption of more functional annotations. For example, although we only explored pig adult tissues, domestication might target biological processes that occur earlier during embryonic development. Thus, generating comprehensive profiles of regulatory signals across multiple developmental stages and tissues would be valuable to further dissect the molecular mechanisms that underlie phenotypic differentiation during domestication. Moreover, by identifying 3D chromosome interactions on a genome-wide scale, Hi-C data would allow for the efficient and accurate assignment of distal signatures of selection to target genes. To compare molecular differences between breeds, we used tissues that are composed of a mixture of different cell subtypes. This type of bulk analysis might have missed differences that are only present in some subtypes, particularly when the proportion of these subtypes is low. Future studies using single-cell technologies to profile the transcriptome and cis-regulatory landscape would allow the exploration of breed differences at a much finer scale, uncovering DNA variants that affect phenotypes through rare but pivotal cell subpopulations.

## Conclusions

In this work, we assembled a high-quality genome of the Chinese indigenous Luchuan pig and identified 26.64 × 10^6^ SNPs and 3.74 × 10^6^ indels from whole-genome resequencing data of 234 pigs, including eastern and western pigs. The genetic differentiation between Luchuan and Duroc pigs provides useful resources for understanding the genetic basis that underlies phenotypic differentiation in pigs. By generating and exploiting multi-tissue gene expression and chromatin accessibility data, our work highlights the importance of non-coding variants in shaping phenotypic differences in pigs and proposes candidate genes and regulatory sequences that might have been subject to selection. By integrating non-coding functional genomic annotations, our work provides a novel perspective to dissect the molecular mechanisms that underlie economic traits in livestock.

## Supplementary Information

**Additional file 1. **Additional materials and methods [20, 21, 24–52, 54–57, 59–66, 118–130]

**Additional file 2: Table S1.** Statistics of the resequencing data produced by this study. **Table S2.** Overview of the data that were downloaded from NCBI. **Table S3.** Statistics of the NCBI data—according to run_ID. **Table S4.** Primers used in this study. **Table S5.** Information on the luciferase reporter assay for 10 selected potential enhancers. **Table S6.** siRNA sequences used in this study. **Table S7.** BioNano optical maps of the Luchuan pig. **Table S8.** Statistics of the two haplotigs in each step of assembly processes. **Table S9.** Size distribution of presence variations in Luchuan assembly compared with Sscrofa11.1 reference. **Table S10.** Predicted protein-coding genes located in the regions of Luchuan pig presence variations compared with the Sscrofa11.1 reference. **Table S11.** GO enrichment of genes in expanded and contracted families of Luchuan pig (qvalue < 0.05). **Table S12.** Statistics of the SNP dataset and Duroc genome. **Table S13.** Cross-validation error value. **Table S14.** Selected genes in eastern pig group. **Table S15.** Selected genes in western pig group. **Table S16.** (a) Enrichment pattern of genes under potential selection in pig GWAS regions; and (b) Enrichment pattern of genes under potential selection in pig QTL regions. **Table S17.** (a) Genes with selective signals, GWAS signals, and highly differential functional coding variants; and (b) Genes with refined selective signals and GWAS signals relevant to pig economic traits. **Table S18.** Information of *LYZ* promoter variants. **Table S19.** Information of the novel lncRNAs. **Table S20.** Differentially expressed genes between Luchuan and Duroc pigs in different tissues. **Table S21.** Statistics of differentially expressed genes including both mRNAs and lncRNAs. **Table S22.** Five types of differential isoform usage between Luchuan and Duroc in each tissue. **Table S23.** Enrichment of DEG promoters in highly differential variants under potential selection. **Table S24.** DEG that do not have highly differential functional coding but promoter variants under potential selection. **Table S25.** ATAC-seq reads and peaks of each sample. **Table S26.** GO enrichment of ATAC-seq peaks in different tissues. **Table S27.** Motif enrichment of ATAC-seq peaks in different tissues. **Table S28.** Enrichment of open chromatin regions with promoters excluded in highly differential variants under potential selection. **Table S29.** Significant enriched GO terms of differential ATAC-seq peaks. **Table S30.** (a) DEG associated with promoters with highly differential variants under potential selection; and (b) DEG associated with distal differential ATAC-seq peaks with highly differential variants. **Table S31.** Identification of putative functional lncRNAs in pig genome by integrative analysis. **Table S32.** Alternative splicing under selection in the ± 2 bp region of the relevant intron/exon junctions.

**Additional file 3: Figure S1.** Overview of Luchuan genome assembly. (a) The flowchart of the contig, scaffold, and chromosome assembly in this study; and (b) Hi-C contact heatmap. Genome-wide analysis of chromatin interactions in Luchuan genome. The numbering of a chromosome is based on the chromosome length from the longest to the shortest. **Figure S2.** Circos plot shows the genome characterization of the Luchuan and Duroc pigs. (I) Syntenic alignments between the Luchuan and Duroc genome based on one-to-one orthologous genes; (II) GC content in non-overlapping 1 Mb windows; (III) Percent coverage of TEs in non-overlapping 1 Mb windows; (IV) Gene density calculated on the basis of the number of genes in non-overlapping 1 Mb windows; and (V) The length of pseudo-chromosome, light red, and light purple ideograms represent the chromosomes of Luchuan and Duroc pigs, respectively. Each ticket above the ideograms is on a scale of 50 Mb. **Figure S3.** Comparative genomic and phylogenetic analyses. (a) Venn diagram showing shared orthologous groups among genomes of Luchuan, Duroc, goat, and human; and (b) Phylogenetic tree with divergence times and history of orthologous gene families. Numbers on the nodes represent divergence times, with the error range shown in parentheses. The numbers of gene families that expanded (red) or contracted (blue) in each lineage after speciation are shown on the corresponding branch. MRCA is the most recent common ancestor. **Figure S4.** PCA plots. (a) PCA of the first three principal components. BAMEI, Bamei; BER, Berkshire; BMX, Bamaxiang; CWB, Chinese wild boar; DUR, Duroc; EHL, Erhualian; GST, Tibetan (Gansu); HAM, Hampshire; HTDE, Hetao; JH, Jinhua; KBP, Korean black pig; KWB, Korean wild boar; LD, Landrace; LUC, Luchuan; LW, Large White; LWH, Laiwu; MEI, Meishan; MIN, Min; PTL, Pietrain; RC, Rongchang; SCT, Tibetan (Sichuan); TC, Tongcheng; TT, Tibetan (Tibet); WZS, Wuzhishan; YM, Yucatan Miniature; YNT, Tibetan (Yunnan); and (b) PCA plots of the first two principal components by classifying them into seven groups: Chinese wild boar (CWB), Korean wild boar (BWB), Luchuan pig (LUC), Duroc (DUR), Korean black pig (KBP), other eastern pigs (EST) and western pigs (WST). **Figure S5.** (a) LD decay determined by squared correlations (r^2^) of allele frequencies against the distance between polymorphic sites in eastern (EST; orange) and western (WST; green) pig groups. (b) LD decay in DUR (Duroc; red) and LUC (Luchuan; blue) pig groups. **Figure S6.** Enrichment pattern of genes under potential selection in GWAS regions associated with some economic traits. Red represents genes located in selective regions of eastern pigs while blue represents those of western pigs. * represents FDR < 0.05. ** represents FDR < 0.01. **Figure S7.** GO enrichment of differentially expressed genes between Luchuan and Duroc pigs in 10 tissues. GO biological process analysis of the differentially expressed mRNAs between Luchuan and Duroc pigs in each tissue. Up indicates the genes with expression levels that are higher in Luchuan pigs compared to Duroc pigs. Down indicates the genes with expression levels that are lower in Luchuan pigs compared to Duroc pigs. **Figure S8.** Analysis of DEG in skeletal muscle tissues. (a) The number of DEG in different tissues. Duroc: the expression level is higher in Duroc pigs compared to Luchuan pigs. Luchuan: the expression level is higher in Luchuan pigs compared to Duroc pigs; (b) Heatmap showing the expression of DEG related to “muscle contraction” in skeletal muscle tissues; and (c) Heatmap showing the expression of DEG related to “cell cycle” in skeletal muscle tissues. **Figure S9.** Frequencies of the Luchuan promoter allele across different pig breeds. DUR: Duroc pig; LW: Large White pig; LD: Landrace pig; YM: Yucatan miniature pig; YNT: Tibetan pig (Yunnan); TC: Tongcheng pig; TT: Tibetan pig (Qinghai-Tibetan); HTDE: Hetaodaer pig; SCT: Tibetan pig (Sichuan); WZS: Wuzhishan pig; BMX: Bamaxiang pig; EHL: Erhualian pig; LUC: Luchuan pig; LWH: Laiwu black pig; MIN: Min pig (Shandong). **Figure S10.** QC of ATAC-seq peaks. Heatmap of reads density around TSS for five tissues. **Figure S11.** Muscle fiber differences between Duroc and Luchuan pigs. Proportions of slow muscle fiber. **Figure S12.** Knockdown and overexpression efficiency of *Tnnc1* in C2C12 cells and in vivo. (a) *Tnnc1* expression after siRNA knockdown in C2C12 cells; (b) *Tnnc1* expression after overexpression in C2C12 cells; and (c) *Tnnc1* expression after in vivo siRNA knockdown in mouse skeletal muscle. **Figure S13.** (a) Expression of *Ho-1* after overexpression of *Tnnc1*; (b) Expression of *Ho-1* after siRNA knockdown of *Tnnc1*; (c) Expression of *Ogg1* after overexpression of *Tnnc1*; and (d) Expression of *Ogg1* after siRNA knockdown of *Tnnc1*. **Figure S14.** (a) Expression of slow muscle (*Myh7*) and fast muscle (*Myh2*, *Myh4*, *Myh1*) markers after siRNA knockdown of *Sema3g* in C2C12 cells; (b) Measuring the same markers as (a) after overexpression of *Sema3g* in C2C12 cells; (c) Choosing siRNAs with the best knockdown efficiency; (d) Evaluation of the overexpression efficiency; and (e) Comparing the expression level of *Sema3g* before and after knocking down *Cebpa*. * represents p-value < 0.05. ** represents p-value < 0.01.

## Data Availability

The Luchuan pig genome assembly and the multi-omics sequencing data are available from the China National GenBank Nucleotide Sequence Archive (CNSA) under accession number CNP0001159 (https://db.cngb.org/search/project/CNP0001159/), and the NCBI database under the accession number PRJNA740359 (https://dataview.ncbi.nlm.nih.gov/object/PRJNA740359?reviewer=m9i6n6f4skv0mvq9vb1qibfc4m).
