# Supplementary material for: Integration of multi-omics data reveals cis-regulatory variants that are associated with phenotypic differentiation of eastern from western pigs
Source: Genet Sel Evol. 2022 Sep 14;54:62. doi: 10.1186/s12711-022-00754-2 (PMC9476355; doi:10.1186/s12711-022-00754-2)
Supplement: Supplementary file 1 — Additional file 1. Additional materials and methods [20, 21, 24–52, 54–57, 59–66, 118–130] [file 12711_2022_754_MOESM1_ESM.docx]

**Additional file 1**

**Additional materials and methods**

**Sample collection**

A male Luchuan boar was obtained from the Institute of Animal Science of Guangxi province, China, for genome assembly. Genomic DNA was extracted from a blood sample. To improve genome annotation, RNA from 14 tissues (heart, lung, subcutaneous adipose, kidney, liver, cerebrum, spleen, stomach, biceps muscle of the thigh, *longissimus dorsi* muscle, testis, ovary, large intestine, small intestine) at four developmental stages (days 0, 14, 50 and adult pigs) from four Luchuan individuals were equally pooled together. However, due to the technical difficulty of collecting some samples, samples were not available for the four developmental time points for all tissues. The total number of tissue by time point combinations was 37.

To analyze differentially-expressed genes between the Luchuan and Duroc breeds, ten tissues were collected from adult Luchuan and Duroc pigs (90-120 kg body weight) for RNA-seq, including skeletal muscle (*longissimus dorsi* muscle), subcutaneous adipose, cerebellum, cerebrum, heart, liver, lung, pancreas, small intestine, and stomach. Five of these tissues (cerebellum, cerebrum, skeletal muscle, small intestine, liver) were subjected to ATAC-seq experiments. Three individuals from each breed were sampled as biological replicates for RNA-seq and two for ATAC-seq, except for pancreas tissue. Tissue samples were manually dissected and then rapidly frozen in liquid nitrogen. The Luchuan and Duroc pigs were raised in the same environment on our pig farm and were sacrificed at a commercial slaughter house. All animal procedures were performed according to protocols approved by the Biological Studies Animal Care and Use Committee in Guangdong Province, China, and guidelines for the Care and Use of Experimental Animals established by the Ministry of Agriculture and Rural Affairs of China.

**Preparation of next-generation sequencing libraries for genome assembly**

In order to generate a chromosome-scale assembly, four genome libraries were constructed and sequenced according to the manufacturers' instructions: (i) whole-genome sequencing (WGS) on the PacBio Sequel platform (20-kb library with SMARTer PCR cDNA Synthesis kit); (ii) Hi-C chromosome conformation captured reads sequencing by Phase Genomics [25] (Hi-C library were prepared with Phase Genomics Proximo Hi-C kit (Animal)); (iii) short reads paired-end sequencing (150-bp paired-end library construction with the Next Ultra DNA library prep kit) by the Illumina NovaSeq 6000 platform; (iv) BioNano optical map data (Nt.BspQI, Nb.BssSI, and DLE-1 enzymes, the library was prepared with the Bionano Prep DLS Labeling kit).

To annotate the Luchuan transcripts, two strand-specific RNA-seq libraries with an insert size of 350 bp were prepared using the NEBNext® UltraTMDirectional RNA Library Prep Kit for Illumina® (NEB, USA) and sequenced on an Illumina NovaSeq 6000 platform, to generate 150-bp paired-end reads (Berry Genomics Co., Ltd., Tianjin, China). A PacBio full-length cDNA library was constructed following the manual of the DNA Template Prep kit (Pacific Biosciences, USA) and sequenced on the Pacific Bioscience RS II sequencer (BerryGenomics, Co., Ltd., Beijing, China).

**Genome assembly with Pacbio data and Hi-C data**

The primary contigs were assembled with the Falcon software packages (v2.0.5) followed by the FALCON-Unzip and Arrow (v2.2.2) polishing, and then a Hi-C-based contig phasing was processed by FALCON-Phase to create phased, diploid contigs. Phase Genomics' Proximo Hi-C genome scaffolding platform was used to create chromosome-scale scaffolds from the draft assembly in a method similar to that described by Bickhart et al. [24]. Following diploid chromosomal scaffolding using Proximo, scaffolds were examined using Juicebox (v1.8.8) [26] to correct small errors in chromosome assignment or contig ordering and orientation to improve scaffold quality. After a draft set of scaffolds was generated, FALCON-Phase was run again for Hi-C-based scaffold phasing. Finally, Pilon (v1.22) was used to correct errors introduced into the assembly as a result of errors in the long reads [27].

The following series of commands were run to correct any remaining phasing errors latent in the draft scaffolds:
1. falcon-phase bamfilt -f 20 -m 10 -i <pseudohap BAM> -o <filtered_pseudohap_bam>
2. falcon-phase bam2 binmat <filtered_pseudohap_bam> <pseudohap_binmat>
3. falcon-phase phase -f <pseudohap FASTA> -b <pseudohap_binmat> -m GATC -i
<scaffold_index> -p <output.phased.txt> -n 10000000 -s 10

**Correction, ordering, and orientation of the initial assemblies by Bionano**

High-molecular-weight DNA was extracted from blood samples using the Bionano Prep Blood DNA Isolation Protocol and digested with Nt.BspQI, Nb.BssSInickase, and DLE-1, respectively. After labeling and staining, DNA was loaded onto the Saphyr chip for sequencing. For each of the three enzyme libraries, respectively 453 Gb, 345 Gb, and 618 Gb of data were collected and converted into a BNX file by the AutoDetect software to obtain basic labeling and DNA length information. The filtered raw DNA molecules in BNX format were aligned, clustered, and assembled into the BNG map using the Bionano Solve pipeline. Two-enzyme (Nt.BspQI and Nb.BssSI) hybrid scaffolding was first processed to produce initial hybrid scaffolds, followed by the second round of hybrid scaffolding with a genome map of the DEL-1 enzyme. Finally, given that a relatively good reference of the Y chromosome was available for the Duroc breed (Sscrofa11.1), a reference-assisted scaffolding strategy was used to obtain chromosome-level pseudomolecules of the Y chromosome with the Chromosomer software (v0.1.4a) [28]. Quality control of the integrity of the assembly was performed by using the independent BUSCO v3 benchmark [29,30], which specifically assesses the integrity of genic regions.

**Detection** **of presence-absence variations between the Luchuan and Duroc genomes and of repeats**

Presence-absence variations (PAV) between the genomes of Luchuan and Duroc pigs were detected by scanPAV with default parameters [31,32]. Short PAV ($\leq1kb$) were filtered as recommended by scanPAV.

Tandem repetitive sequences were identified using the Tandem Repeats Finder (version 4.07) software. The interspersed repeat contents of the Luchuan pig genome were identified using *de novo* repeat identification and known repeat searching against existing databases. RepeatModeler (version 1.0.8) was used to predict repeat sequences in the genome. RepeatMasker (version 4.0.7) was then used to search the Luchuan pig genome against the *de novo* transposable element (TE) library [33]. The homology-based approach involved the use of commonly used databases of known repetitive sequences, and the RepeatMasker (version 4.0.7) software and Repbase database (version 21) were used to identify TE in the assembled genome.

**Gene prediction and annotation**

Protein-coding region identification and gene prediction were conducted through a combination of homology-based prediction, *de novo* prediction, and transcriptome-based prediction methods. Proteins from six mammal genomes (human, mice, cattle, dogs, goats, and Duroc pigs) were downloaded from ensemble release-95. Protein sequences were aligned to the assembly using the TBLASTN program available in the BLAST v2.2.24 (E value cutoff 1E−5), then SOLAR (Sorting Out Local Alignment Results) version0.9.6, a dynamic programming algorithm to link putative exons together, was employed to analyze the TBLASTN results. GeneWise (version 2.4.1) was used to predict the exact gene structure of the corresponding genomic regions on each matched sequence. Four ab initio gene prediction programs, Augustus (version 3.2.1), GlimmerHMM (version 3.0.4), Geneid (version 1.4.4), and SNAP (version 2006-07-28), were used to predict coding regions in the repeat-masked genome. Finally, RNA-seq data were mapped to the assembly using hisat2 (version 2.0.1), stringtie (version 1.2.2), and TransDecoder (version 3.0.1) were then used to assemble the transcripts and identify candidate coding regions into gene models. Transcripts in PacBio single-molecule sequencing data were identified by IsoSeq3 (version 3.1.0) with default parameters, and then the Iso-Seqdatas were mapped to reference genome with minimap2 (version 2.15-r905), Cupcake ToFU (v5.8) was used to get the final unique, full-length, high-quality isoforms of Pacbio data. All gene models predicted from the above three approaches were combined by EvidenceModeler (EVM) into a non-redundant set of gene structures, and the produced gene models were finally refined with the Program to Assemble Spliced Alignments (PASA v2.3.3). Functional annotation of protein-coding genes was achieved using BLASTP (E-value 1e-05) against two integrated protein sequence databases: SwissProt and TrEMBL. Protein domains were annotated by using InterProScan (V5.30). The Gene Ontology (GO) terms for each gene were extracted with InterProScan. The pathways in which the genes might be involved were assigned by BLAST against the KEGG databases (release 59.3), with an E-value cutoff of 1e-05.

**Prediction of non-coding RNA annotations based on the Luchuan genome assembly**

The tRNA genes were predicted by tRNAscan-SE (version 1.3.1). The rRNA fragments were predicted by aligning to human template rRNA sequences using BlastN (version 2.2.26) at an E-value of 1e-5. The miRNA and snRNA genes were found by searching against the Rfam database (release 12.0) using INFERNAL (version 1.1.1). Long non-coding RNAs (LncRNAs) and Circular RNAs (circRNAs) were predicted by methods described previously [20, 34, 35].

**Identification of gene families**

A gene family indicated a set of similar genes that descended from a single original gene in the last common ancestor of considered species. Orthologous gene sets of Luchuan pigs, Duroc pigs, cattle, goats, dogs, mice, and humans were used for genome comparisons. For genes with alternative splicing variants, we chose the longest transcripts (≥ 50 amino acids) to represent the genes. BLASTP was applied to all protein sequences using a database containing protein data set from the seven mammalian genomes, and the TreeFam methodology [36] was used to define a gene family and resulted in 3,733 single-copy orthologous genes for the seven mammalian species.

**Construction of a phylogenetic tree and estimation of divergence time among different mammal species**

The 3,733 single-copy gene families described above were used to construct a phylogenetic tree for Luchuan pigs and the other mammalian genomes (Duroc pigs, cattle, goats, dogs, mice, and humans). Four-fold degenerate sites were extracted from each family and concatenated to form one supergene for each species. The GTR+gamma substitution model was selected, and PhyML v3.0 [37] was used to reconstruct the phylogenetic tree. The divergence time among Luchuan pigs, Duroc pigs, cattle, goats, dogs, mice, and humans were estimated using the MCMCtree program (version 4.4) as implemented in the Phylogenetic Analysis of Maximum Likelihood (PAML) package, with an independent rates clock and HKY85 nucleotide substitution model. The calibration times (differentiation time between humans and mice, humans and goats, cattle and goats, pigs and goats) were derived from the TimeTree database [38]. Changes in gene family size along the phylogenetic tree were analyzed by CAFE (v2.1) [39].

**Library preparation for population-based resequencing and SNP calling**

Genomic DNAs from the ear tissues of 16 Luchuan pigs, 18 Tongcheng pigs, and 38 Large White pigs were extracted using the DNeasy Blood & Tissue Kit (Qiagen) according to the manufacturer's instructions. Libraries were made according to the manufacturer's standard protocols (Illumina). Sequencing was performed to generate 150-bp paired-end reads on the Illumina NovaSeq 6000 platform (Berry Genomics Co., Ltd., Tianjin, China). The sequencing data for each individual reached more than 35-fold depth [see Additional file 2 Table S1]. In addition, we downloaded the genome sequencing data of 157 western and eastern pigs with the lowest sequencing depth of more than 10 X. The total downloaded data reached 8.56 Tb with an average depth of 21.14 X (genome size calculated according to 2.50 G) [20, 40, 41, 21] [see Additional file 2 Table S2 and S3].

The high-quality paired-end reads were mapped to the Duroc (Sscrofa11.1) reference genome using BWA (v0.7.12) [42] with the parameter: 'mem -t 4 -k 32 –M'. PCR or optical duplicates were removed using SAMtools (v1.3.1) [118]. We performed SNP calling using a UnifiedGenotyper approach as implemented in the package GATK (Genome Analysis Toolkit, v3.7-0-gcfedb67). To remove the potential false-positive SNPs, SNPs with QD < 2.0 or FS > 60.0 or MQ < 20.0 or MQRankSum < –12.5 or ReadPosRankSum < –8.0 were filtered.

Gene-based SNP annotation was performed according to the annotation Sscrofa11.1.94 of the Duroc genome using the package ANNOVAR (v2013-06-21) [43, 44]. Based on the genome annotation, SNPs were categorized as occurring in exonic regions (overlapping with a coding exon), intronic regions (overlapping with an intron), splicing sites (within 2 bp of a splicing junction), upstream and downstream regions (within a 1 kb region upstream or downstream from the transcription start site), or intergenic regions.

**Evaluation of SNP depletion in coding sequence regions**

The number of SNPs in coding sequence (CDS) regions was recorded as Osnp. To determine whether SNPs are significantly depleted in CDS regions, we performed 1000 rounds of simulation to mimic the random distribution of SNPs in CDS regions. For each round of the simulations, we sampled genomic intervals from the pig genome (susScr11) excluding repeats annotated by "RepeatMasker" to match the number and length of the set of merged CDS regions and counted the number of SNPs that overlapped with these intervals, recorded as Ssnp. The P-value for whether SNPs are significantly depleted in CDS regions was determined by the proportion that Ssnp was smaller than Osnp.

**Phylogenetic and population genetic analyses**

To analyze the population structure, we screened a subset of bi-allelic and high-quality SNPs with a call rate ≥ 90% and a minor allele frequency (AF) ≥ 5%. A neighbor-joining (NJ) tree was constructed using the program TreeBeST (v1.92) with 200 bootstrap replicates [45]. The tree was displayed using MEGA [46]. To infer the population structure, we used ADMIXTURE (v1.3.0) [47], which implements a block-relaxation algorithm. To make consideration for Hardy-Weinberg equilibrium (HWE) violations, we also filtered SNPs by testing HWE violations (P > 10^−4^) and reconstructed the model-based clustering analysis. To identify the best genetic clusters K, cross-validation error was tested for each K value from 2 to 10. The termination criterion was 10^–6^ (stopping when the log-likelihood increased by less than ε = 10^–6^ between iterations). We also performed principal component analysis (PCA) using the program GTAC (v1.92) [48].

**Linkage disequilibrium analysis**

To estimate and compare the pattern of linkage disequilibrium (LD) of domesticated breeds, the squared correlation coefficient (r^2^) values between any two SNPs within 300-kb intervals were computed by using the Haploview (v4.269) software [49]. We produced an LD decay plot that shows the average r^2^ values in 100-bp bins against the physical distance of pairwise bins. To get reliable results, wild boars (Chinese wild boars [CWB], Korean wild boars [KWB]) and Korean black pigs [KBP], a breed with an uncertain genetic background and that is mixed with Western lineages according to the admixture results, were excluded from the LD analysis and from the subsequent selective sweep analyses.

**Analysis of selective sweeps**

We used multiple methods to detect regions and genes under selection. For a genomic locus, the selection is expected to increase genetic differentiation (F_ST_) between populations and to reduce nucleotide diversity (θπ) in the population in which the selective sweep occurs. SNPs with a MAF lower than 5% were removed from this analysis. Estimates of θπ and F_ST_ of Eastern and Western pig populations were calculated using the VCFtools (v0.1.13) package [50] with a 50-kb sliding window and a step size of 10 kb. Windows that contained less than 10 SNPs were excluded from further analysis [51]. Windows that were both in the top 10% of F_ST_ values and in the 5% left or right tails of the empirical ratio (θπ Eastern/θπ Western) regions were identified as regions under selection in Eastern and Western pigs. In addition, to avoid missing fixed signatures of selection that have both small θ_π_ values in each population and high between-population F_ST_, windows that were both in the bottom 5% of θ_π_ in each population and in the top 10% of F_ST_ were also considered as regions under selection. We combined all these regions into a set of putative regions under selection. The figure was drawn using RectChr (Version 1.24) [52]. GO enrichment analysis of genes under selection was implemented with the GOseq R package [53]. GO terms with corrected P-values < 0.05 were considered to be significantly enriched.

**Enrichment analysis of genes under potential selection in pig QTL/GWAS regions**

Phenotype-associated loci derived from pigQTLdb (updated on April 26th, 2021) [54,55] were partitioned from QTL and GWAS. We collected all genes within the full length of QTL regions, and within 2 Mb genomic regions centered at the midpoints of GWAS signals [56-58], respectively. Then, we used a hypergeometric test to determine whether the genes associated with a trait in QTL/GWAS analysis were enriched in regions under potential selection. Specifically, we calculated p values by $1-\sum_{i=0}^{k-1} \frac{\binom{M}{i}*\binom{N-M}{n-i}}{\binom{N}{n}}$, where $N$ represented the number of all genes located in all QTL/GWAS intervals, $M$ represented in the $N$ genes, the number of all genes with potential selective signals, $n$ represented the number of all genes located in the regions of a specific QTL/GWAS trait, and $k$ represents in the $n$ genes, the number of genes with potential selective signals. The Enrichment was determined by $\frac{k/n}{M/N}$. Finally, we used FDR to adjust the p-value. Terms with an FDR < 0.05 would be considered significant.

**mRNA, lncRNA, and alternative splicing analysis of RNA-seq**

A total of 57 strand-specific RNA-seq libraries with an insert size of 350 bp were prepared using the total RNA of 10 tissues (skeletal muscle, adipose, cerebellum, cerebrum, heart, liver, lung, pancreas, small intestine, and stomach) of Duroc and Luchuan pigs according to the manufacturer's instructions (Illumina, SanDiego, CA). The libraries were sequenced on an Illumina HiSeq 4000 platform to generate 150 bp paired-end reads (Novogene Co., Ltd., Tianjin, China). After removing reads containing adapters, reads containing poly-N, and low-quality reads from the raw data. The RNA-seq clean reads were aligned to the Duroc reference genome by Tophat v2.1.0 with a parameter of --library-type fr-firststrand. Other parameters were set as default [119]. The expression level of genes was quantified by calculating reads per kilobase per million reads (RPKM) values using HTSeq v0.6.1 [120]. LncRNAs were identified using the pipeline in our previous study [121]. Differentially expressed genes between Duroc and Luchuan pigs for each tissue were identified using the cutoffs of FDR ≤ 0.05 and |log2 FC| ≥ 1 by DEseq2 v1.20.0 in R [122].

Alternative splicing (AS) was detected with the RNA-seq data in the above tissues. The RNA-seq data was aligned with STAR [123], and the output bam files were fed to rMATS to investigate AS events [124]. With a cutoff of FDR < 0.05 and |IncLevelDifference| > 5%, five types of differential alternative splicing (DAS) were detected, including alternative 5' splice site (A5SS), alternative 3' splice site (A3SS), mutually exclusive exon (MXE), skipped exon (SE) and retained intron (RI), in which more SE and MXE DAS were found. We subsequently integrated the information of ASGs, SNPs, selected genes (SGs), and selected regions to find the overlapped genes. Finally, only the genes with significant isoform alteration between the two breeds and have at least one high deltaAF (△AF > 0.5) SNP under selection in the ±2 bp region of the relevant intron/exon junctions were kept.

**Western blot**

Proteins were extracted from small intestine tissue in T-PER™ Tissue Protein Extraction Reagent (Thermo Scientific™, USA) with two pieces of Protease inhibitor tablet (Roche, USA). The total protein concentration was quantified using the BCA protein assay kit (Invitrogen). Each sample was separated in a 7.5% SDS-PAGE gel (Invitrogen) and transferred onto a nitrocellulose membrane. The membrane was blocked by incubating for 2h at room temperature with 5% Non-fat milk powder. The membrane was then incubated at 4℃ overnight with a primary antibody, followed by a secondary antibody for 2h at room temperature. These antibodies were diluted with 1% TBST ([Tris-Buffered Saline Tween-20) recipe](https://www.baidu.com/link?url=vW_MPG0V-wqnwBLhzr_Gt8GVN0wGul0oa1i9LwwB5YGmpIu6beSfoOm_1Lasftypy_poq0CTxQv7BK96imq2z_&wd=&eqid=fa7ed99700111194000000025e528ead)). After washing, the bands were visualized with an enhanced chemiluminescence system. Densitometric quantification of the western blot bands was performed using ImageJ software. The primary antibodies included LYZ (PA5-16668; 1:1000, Thermo Scientific™, USA), and GAPDH (FD0063; 1:5000; Fdbio science, China). The secondary antibody is VHH anti-Mouse-HRP (Kangti Life Technology Co., Ltd, China). The protein levels were normalized to those of the protein GAPDH. Densitometric analysis of bands was performed using ImageJ software.

**ATAC-seq analysis**

ATAC-seq was performed as previously described [59]. Basically, samples were homogenized in cold nuclei lysis buffer (copy buffer composition from paper) and ~50,000 nuclei were resuspended in 2xTD buffer from the Illumina Nextera Kit. Transposition was performed at 37℃ for 60 min with gentle shaking. DNA was purified with the Qiagen DNA miniElute column and amplified for 16 cycles with indexed S5 and N7 primers. The final library was size-selected for products between 200-600 bp using the AMPure XP bead and subjected to pair-end sequencing (PE150) on a Novaseq6000 platform.

The ATAC-seq data were processed as previously described [60, 61], with two biological replicates collected for each tissue. The FASTQ sequencing files from ATAC-seq experiments were trimmed with NGSQCToolkit (v2.3.3), then mapped to the *Sscrofa11.1* genome and Luchuan genome with Bowtie2 (v2.2.9) [60], respectively. We took advantage of two good genome assemblies for mapping in order to reduce the mapping bias by differential DNA variants between the two breeds. Reads with high-mapping quality scores (MAPQ > 30) and uniquely alignment was filtered out by Samtools (v1.9). The PCR duplication reads were removed by using Picard (v2.20.4) [125]. For peak calling of open chromatin, reads were filtered using the Deeptools (v3.3.0) command 'alignmentSieve.py' with options –ATACshift [126]. The filtered BAM files were inputted to Fseq (v1.84) at default parameters for peak calling [127]. Reproducible peaks between two biological replicates were identified by the Irreproducible Discovery Rate (IDR) method, and only peaks reproducible were retained for downstream analyses. The reproducible peaks were then used to get fasta sequences from the genome of the two pigs respectively through Bedtools (v2.17.0) [128], and then mapped to the other genome to get the SAM file with bwa (v0.7.17) [129]. Using the SAM file, we got the peak position in both genomes. The number of raw reads mapped to each peak was counted using BedToolsmulticov (v 2.17.0) [49]. We treated each peak as if it was a gene, and identified differential peaks with DESeq2 (v1.22.2) [122]. We performed motif analysis by Fimo (v4.12.0) to find enriched DNA binding motifs of transcription factors in peaks [62].

To explore the enrichment pattern of differential ATAC-seq peaks, we first converted these regions to hg38 coordinates using the LiftOver tool with the parameter "minMatch = 0.1" [63]. After that, we performed enrichment analysis using GREAT [64, 65].

**Analysis of genomic enrichment in regions under selection**

To evaluate the enrichment of DEG promoters of 10 tissues in selected regions, we set promoters of all protein-coding genes as a baseline group. We excluded repeats annotated by "RepeatMasker" as these repeat regions were also excluded when identifying genetic differentiation regions. To avoid spurious enrichment caused by functional coding variants, we removed selected regions that are within 50kb of functional coding variants between Luchuan and Duroc. We counted the observed value (Obs) as the number of highly differential variants between Luchuan and Duroc under selection overlapping promoters. After that, 1000 rounds of simulations were performed. For each round, we sampled genomic intervals to match the number and length of the set of promoters and counted the number of variants mentioned above overlapping each set of intervals, recorded as Sim. We calculated Rand as the average of Sim over 1000 simulations. Finally, the fold of enrichment was calculated as Obs/Rand. P-value was determined by the number of times that Sim >= Obs, divided by 1000.

To evaluate the enrichment of ATAC-seq peaks in selected regions, we first removed ATAC-seq peaks overlapping with promoter regions, and then exploited the similar simulation strategy as in the promoter enrichment analysis.

**Luciferase reporter assays**

To generate luciferase reporter constructs for LYZ promoter, we cloned LYZ gene promoters (1101 bp upstream of TSS) of Luchuan and Duroc, respectively, into pGL3-Basic (Promega, USA) vector between Xho I and Sac I sites by using the Homologous Recombination Kit (Qingke, China). The small intestine cells were cultured at 37°C with Dulbecco's modified Eagle's medium/F-12 (Thermo Fisher), 10% FBS (Gibco, USA), 1% penicillin/streptomycin (Gibco, USA), and 5% CO2. These cells were grown to 75% to 80% confluence in 12-well plates and then the pGL3-Luchuan-LYZ-promoter, pGL3-Duroc-LYZ-promoter, pGL3-Duroc-LYZ-modified-promoter, and pGL3-Basic (Promega, USA) vectors were co-transfected with pRL-TK vector (Promega, USA), respectively. The co-transfection assays were performed in 12-well plates with Lipofectamine 3000 reagent (Invitrogen, China) according to the manufacturer's instructions. Cells were harvested after 24hr followed by a Dual-luciferase assay system (Promega, USA) to examine the activity of luciferase. Primer sequences are in Table S4 [see Additional file 2 Table S4].

To generate luciferase reporter constructs for potential enhancers, we first randomly chose 10 skeletal muscle DEGs using sample() function without replacement in the R program and then attempted to test the associated ATAC-seq regions (differential ATAC-seq peaks with highly differential variants in the skeletal muscle between Luchuan and Duroc) within 1 Mb of these genes in luciferase experiments. For a long peak with multiple highly differential variants, we tested a short DNA fragment in luciferase experiments centering the variant closest to the middle of the peak. These potential enhancer regions were cloned into luciferase reporter vectors [see Additional file 2 Table S5]. These constructs were tested for their enhancer activity by dual luciferase experiments in C2C12 cells, which were grown to 75% to 80% confluence in 12-well plates and then co-transfected with (peak)-pcDNA4.23 vector and PRL-TK using Lipofectamine 3000 reagent (Invitrogen), according to the manufacturer's instructions. The cells were harvested after 24 hours, and luciferase activity was evaluated using a dual-luciferase assay system (Promega).

**Phenotypic analysis of skeletal muscle**

Muscle slow/fast fiber compositions were obtained using the myofibrillar ATPase staining method and microscope counting [66]. In brief, the sampled 10 Landrace and 10 Luchuan longissimus dorsi muscles postmortem were cut into 0.5 × 0.5 × 1.0 cm pieces and promptly frozen by liquid nitrogen. Serial transverse muscle sections (10 μm) of each sample were obtained and mounted on slide glasses. Myosin heavy chain (MYHC) ATPase staining was performed at pH 10.4 for 15mins. Under these alkaline conditions, ATPase in MYHC1 isoforms (slow fibers) was inactivated while in MYHC2b isoforms (fast fibers) were still functional, resulting in heavier staining of the fast muscle fibers. After being washed with 1% calcium chloride three times, the histochemical images of all samples were analyzed by an optical image analysis program [130]. Approximately 300 fibers per sample, which were free from tissue disruption and freeze damage, were evaluated and classified into fiber types based on MYHC1 and MYHC2b staining. The percent of the slow fiber was estimated as the ratio of MYHC1 isoforms fiber and the total number of fibers counted.

The microstructural changes of skeletal muscle fibers in Landrace and Luchuan were evaluated by scanning electron microscopy using Zeiss DSM 962 (Inspect‐F; FEI, Hillsboro, Oregon, USA) under an acceleration voltage of 2 kV and the in-lens secondary electron detector. Samples of LD from Landrace and Luchuan pigs were placed in 3% (vol/vol) glutaraldehyde in PIPES buffer (100 mM, pH 7.4). The hydrogel samples were dehydrated and dried under a vacuum. Then the dried gel was cut, and the cross‐section was sprayed with platin/palladium for SEM viewing.

**RNA interference and overexpression**

The synthetic siRNAs were all obtained from Genepharma (Shanghai, China; [see Additional file 2 Table S6]). The siRNAs knockdown and overexpression of *Tnnc1* were performed in mouse C2C12 cells and the expression levels of *Ho-1* and *Ogg1* were measured. The siRNAs that target *NR2F2* and the NC-siRNA were transfected into pig small intestine cells to measure the expression levels of *LYZ* after *NR2F2* knockdown. The siRNAs that target the *Tnnc1* and *Sema3g* genes, and the NC-siRNA, pcDNA3.1-Tnnc1, pcDNA3.1-Sema3g, and pcDNA3.1-Control were transfected into C2C12 cells to detect expression levels of *Myh7*, *Myh2*, *Myh4*, and *Myh1* in knockdown and overexpression experiments. To generate a *Tnnc1* overexpression vector, the 498-bp coding sequence region of the pig *TNNC1* gene was amplified using forward and reverse primers that contained BamH I and Xho I sites, respectively. The PCR products were inserted into the pcDNA3.1 (+) vector (Invitrogen, China; [see Additional file 2 Table S4]).

**Quantitative real-time polymerase chain reaction**

Total RNA was extracted from small intestine cells using TRIzol reagent (Invitrogen, China), according to the manufacturer's instructions, and then reverse-transcribed to complementary DNA using HiScript III 1st Strand cDNA Synthesis Kit (Vazyme Biotech, China). qRT-PCR was conducted using the SYBR Green Master Mix (Vazyme Biotech, China), and the results were analyzed using the 2△△CT method. Primer sequences are listed in Table S4 [see Additional file 2 Table S4].

**AAV-mediated in vivo knockdown of *Tnnc1* in mice**

7-week-old mice were obtained from the company (Huafukang, Beijing, China). AAV9 serotypes of siRNA-Tnnc1 and siRNA-NT (non-target control) were produced. We injected 1X1012 GCs (genome copies) 100ul of AAV expressing either siRNA-Tnnc1 or siRNA-NT. The anterior tibial muscle in one mouse's right leg was injected with siRNA-Tnnc1. The other mouse tibial anterior muscle of the right leg was injected with siRNA-NT. Mice were anesthetized with isoflurane and killed by cervical dislocation to harvest muscle. Groups of three mice were killed after 14 days to collect the TA muscles.

After 14 days of injection with siRNA-Tnnc1 and siRNA-NT packaged with AAV9 serotype, the TA muscle of mice was collected, and the tissues were soaked in 30% sucrose solution overnight for dehydration. After OCT embedding, frozen sections were fixed in 4% paraformaldehyde overnight, and ATPase staining was carried out according to the alkaline Staining Kit (Solarbio, Beijing, China). The deeper the cross-sectional area of muscle fiber was stained, the stronger the ATPase activity was. The darker color thus indicated a higher proportion of fast muscle fibers.

**Statistical Analysis for qPCR**

SPSS v20.0 (SPSS Inc, Chicago, Illinois) software was used for statistical analysis. T-tests and analysis of variance were used to assess statistical significance. A value of P <0.05 was considered statistically significant. All experiments were repeated 3 times, and all data are expressed as mean + SEM.

**Sequence and function conservation analysis of cis-regulatory elements**

The differential ATAC-seq peaks between Luchuan and Duroc were defined as pig differential cis-regulatory elements (diffCREs). We used liftOver to convert the diffCREs (extended 500 bp both ways from the middle points, susScr11) to the human genome (hg38) with the parameter "minMatch = 0.5" [63,67]. Their homologous human sequences were considered as sequence conserved diffCREs. Furthermore, a diffCRE from a pig tissue would be considered functionally conserved if its human counterpart has DNase signals in the concordant human tissue (ENCODE v5). To investigate the characteristics of these functionally conserved sequences, we performed GO biological process analysis using GREAT [64, 65]. In addition, we perform GWAS analysis by overlapping these functionally conserved homologous sequences with human GWAS signals (50 kb genomic regions centered at GWAS tag SNPs to account for linkage disequilibrium).
