# Supplementary material for: Integration of multi-omics data reveals cis-regulatory variants that are associated with phenotypic differentiation of eastern from western pigs
Source: Genet Sel Evol. 2022 Sep 14;54:62. doi: 10.1186/s12711-022-00754-2 (PMC9476355; doi:10.1186/s12711-022-00754-2)
Supplement: Supplementary file 3 — Additional file 3: Figure S1. Overview of Luchuan genome assembly. (a) The flowchart of the contig, scaffold, and chromosome assembly in this study; and (b) Hi-C contact heatmap. Genome-wide analysis of chromatin interactions in Luchuan genome. The numbering of a chromosome is based on the chromosome length from the longest to the shortest. Figure S2. Circos plot shows the genome characterization of the Luchuan and Duroc pigs. (I) Syntenic alignments between the Luchuan and Duroc genome based on one-to-one orthologous genes; (II) GC content in non-overlapping 1 Mb windows; (III) Percent coverage of TEs in non-overlapping 1 Mb windows; (IV) Gene density calculated on the basis of the number of genes in non-overlapping 1 Mb windows; and (V) The length of pseudo-chromosome, light red, and light purple ideograms represent the chromosomes of Luchuan and Duroc pigs, respectively. Each ticket above the ideograms is on a scale of 50 Mb. Figure S3. Comparative genomic and phylogenetic analyses. (a) Venn diagram showing shared orthologous groups among genomes of Luchuan, Duroc, goat, and human; and (b) Phylogenetic tree with divergence times and history of orthologous gene families. Numbers on the nodes represent divergence times, with the error range shown in parentheses. The numbers of gene families that expanded (red) or contracted (blue) in each lineage after speciation are shown on the corresponding branch. MRCA is the most recent common ancestor. Figure S4. PCA plots. (a) PCA of the first three principal components. BAMEI, Bamei; BER, Berkshire; BMX, Bamaxiang; CWB, Chinese wild boar; DUR, Duroc; EHL, Erhualian; GST, Tibetan (Gansu); HAM, Hampshire; HTDE, Hetao; JH, Jinhua; KBP, Korean black pig; KWB, Korean wild boar; LD, Landrace; LUC, Luchuan; LW, Large White; LWH, Laiwu; MEI, Meishan; MIN, Min; PTL, Pietrain; RC, Rongchang; SCT, Tibetan (Sichuan); TC, Tongcheng; TT, Tibetan (Tibet); WZS, Wuzhishan; YM, Yucatan Miniature; YNT, Tibetan (Yunnan); and (b) PCA plots of t [file 12711_2022_754_MOESM3_ESM.pdf]

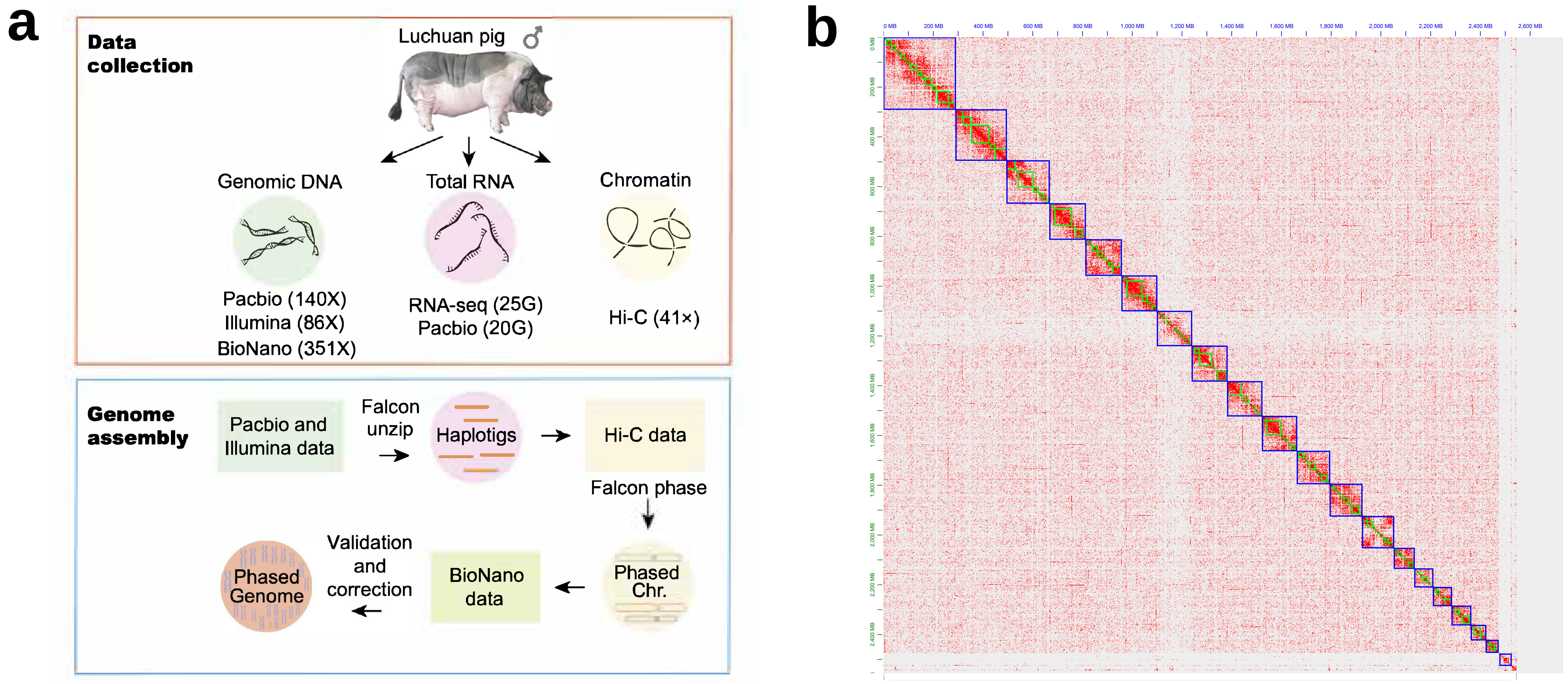

Figure S1. Overview of Luchuan genome assembly.
(a) The flowchart of the contig, scaffold, and chromosome assembly in this study. (b) Hi-C contact heatmap. Genome-wide analysis of chromatin interactions in Luchuan genome. The numbering of a chromosome is based on the chromosome length from the longest to the shortest.

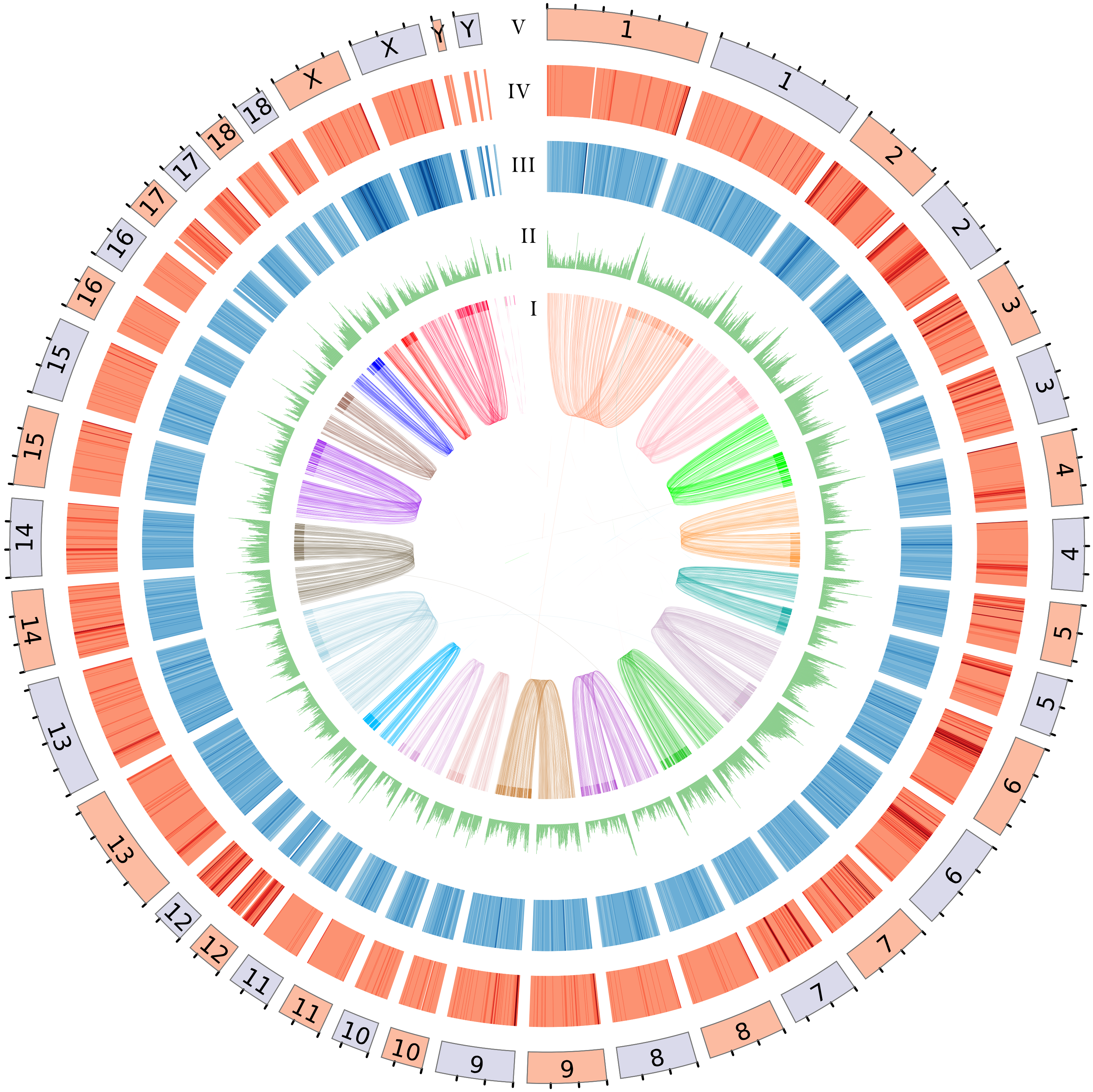

Additional file 2 Figure S2. Circos plot shows the genome characterization of the Luchuan and Duroc pigs.
I: Syntenic alignments between the Luchuan and Duroc genome based on one-to-one orthologous genes.
II: GC content in non-overlapping 1 Mb windows.
III: Percent coverage of TEs in non-overlapping 1Mb windows.
IV: Gene density calculated on the basis of the number of genes in non-overlapping 1Mb windows.
V: The length of pseudo-chromosome, light red, and light purple ideograms represent the chromosomes of Luchuan and Duroc pigs, respectively. Each ticket above the ideograms is on a scale of 50 Mb.

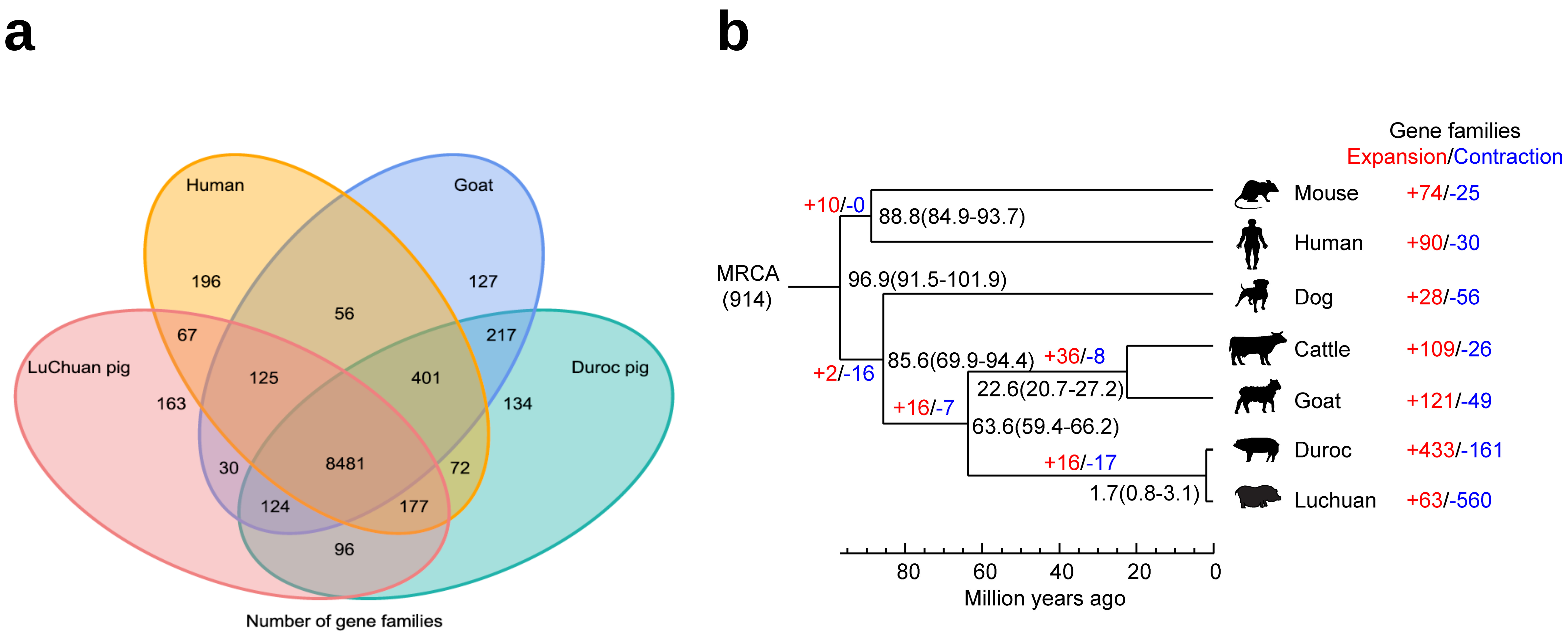

Figure S3. Comparative Genomic and Phylogenetic Analyses.
(a) Venn diagram showing shared orthologous groups among genomes of Luchuan, Duroc, goat, and human. (b) Phylogenetic tree with divergence times and history of orthologous gene families. Numbers on the nodes represent divergence times, with the error range shown in parentheses. The numbers of gene families that expanded (red) or contracted (blue) in each lineage after speciation are shown on the corresponding branch. MRCA is the most recent common ancestor.

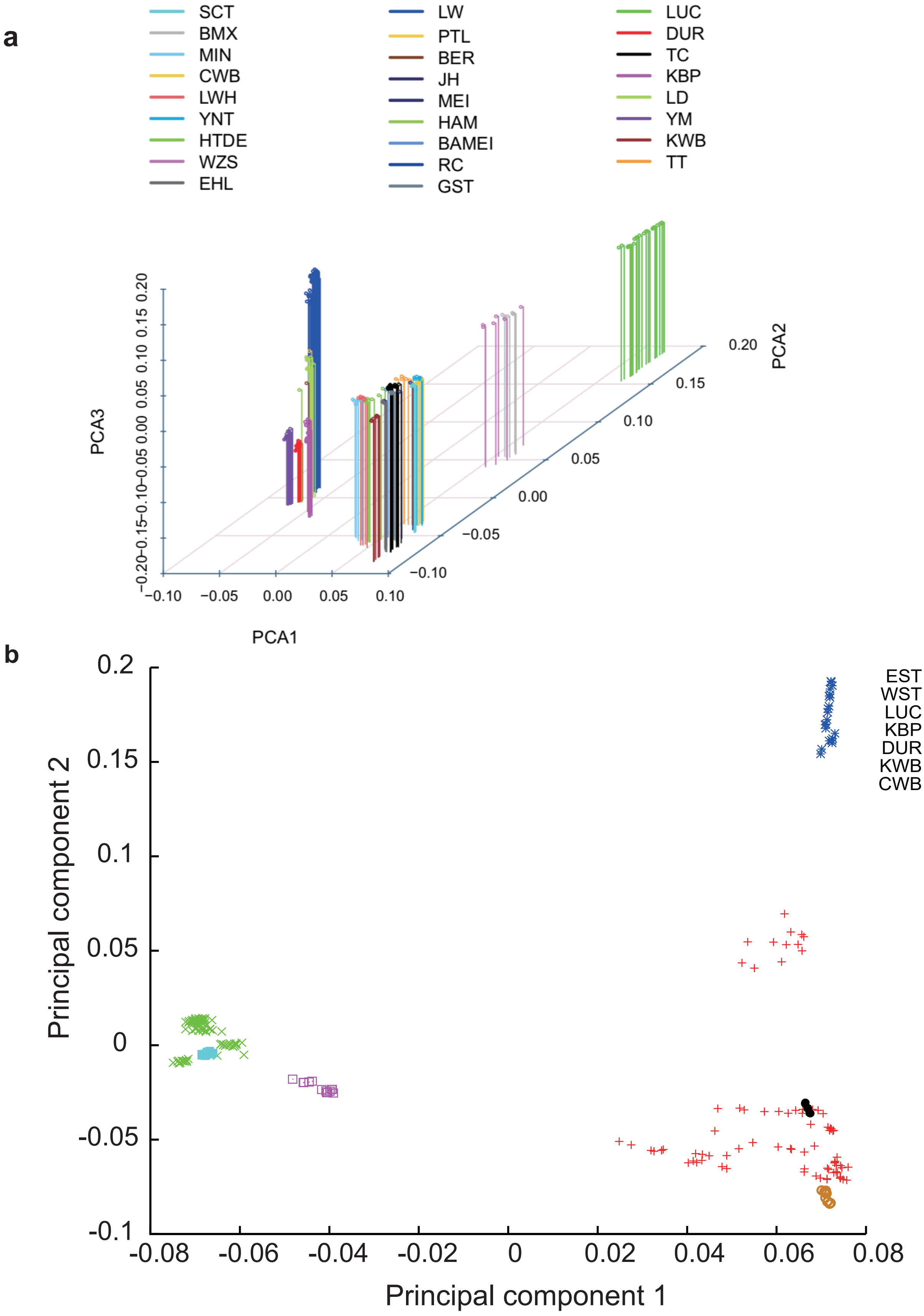

Figure S4. PCA plots. (a) PCA of the first three principal components. BAMEI, Bamei; BER, Berkshire; BMX, Bamaxiang; CWB, Chinesewild boar; DUR, Duroc; EHL, Erhualian; GST, Tibetan (Gansu); HAM, Hampshire; HTDE, Hetao; JH, Jinhua; KBP, Korean black pig; KWB, Korean wild boar; LD, Landrace; LUC, Luchuan; LW, Large White; LWH, Laiwu; MEI, Meishan; MIN, Min; PTL, Pietrain; RC, Rongchang; SCT, Tibetan (Sichuan); TC, Tongcheng; TT, Tibetan (Tibet); WZS, Wuzhishan; YM, Yucatan Miniature; YNT, Tibetan (Yunnan). (b) PCA plots of the first two principal components by classifying them into seven groups: Chinese wild boar (CWB), Korean wildboar (BWB), Luchuan pig (LUC), Duroc (DUR), Korean black pig (KBP), other Eastern pigs (EST) and Western pigs (WST).

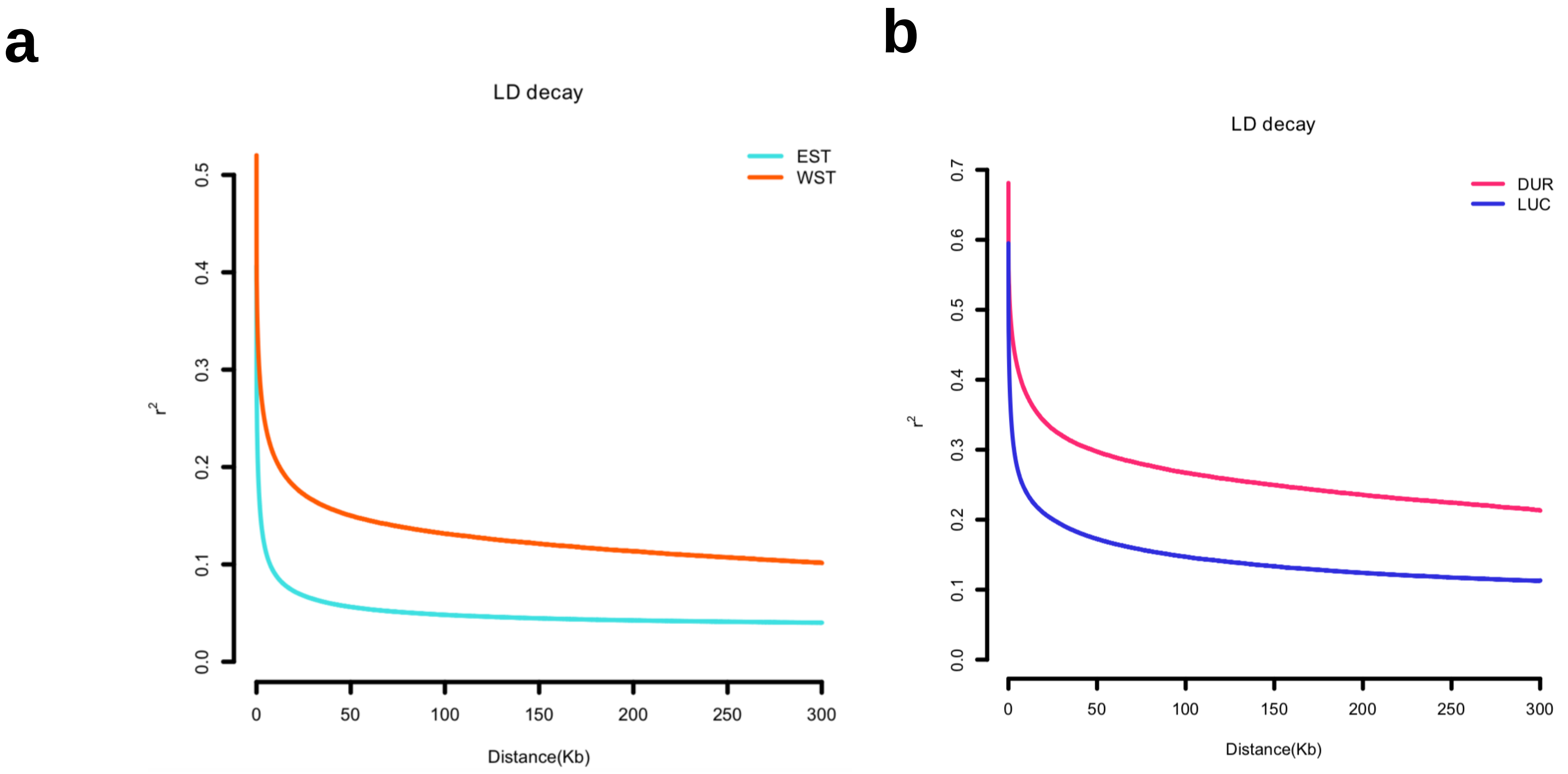

Figure S5. LD decay analysis. (a) LD decay determined by squared correlations (r^2) of allele frequencies against the distance between polymorphic sites in eastern (EST; orange) and western (WST; green) pig groups. (b) LD decay in DUR (Duroc; red) and LUC (Luchuan; blue) pig groups.

Figure S6. Enrichment pattern of genes under potential selection in GWAS regions associated with some economic traits. Red represents genes located in selective regions of eastern pigs while blue represents those of western pigs. * represents FDR < 0.05. ** represents FDR < 0.01.

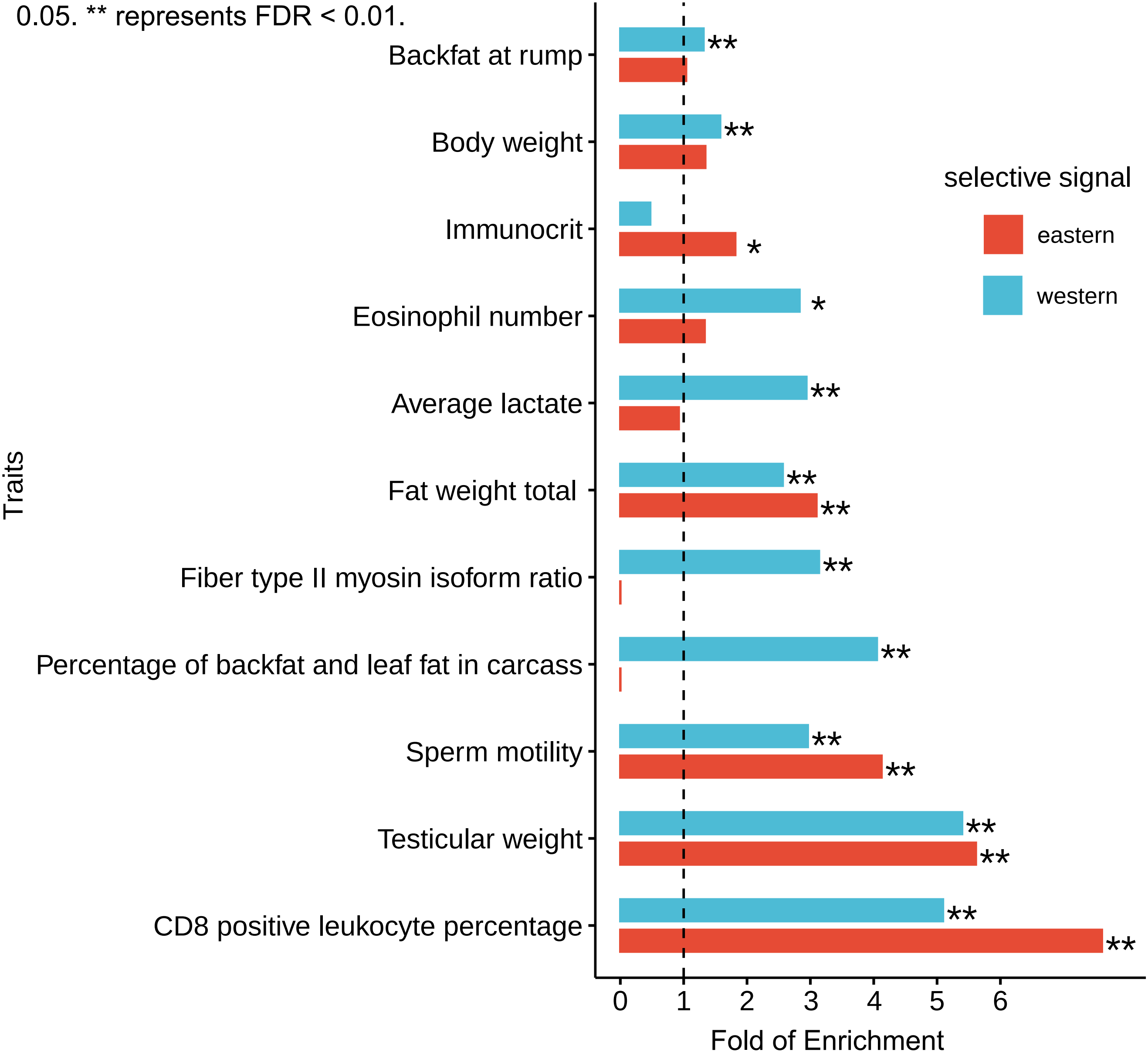

Figure S7.

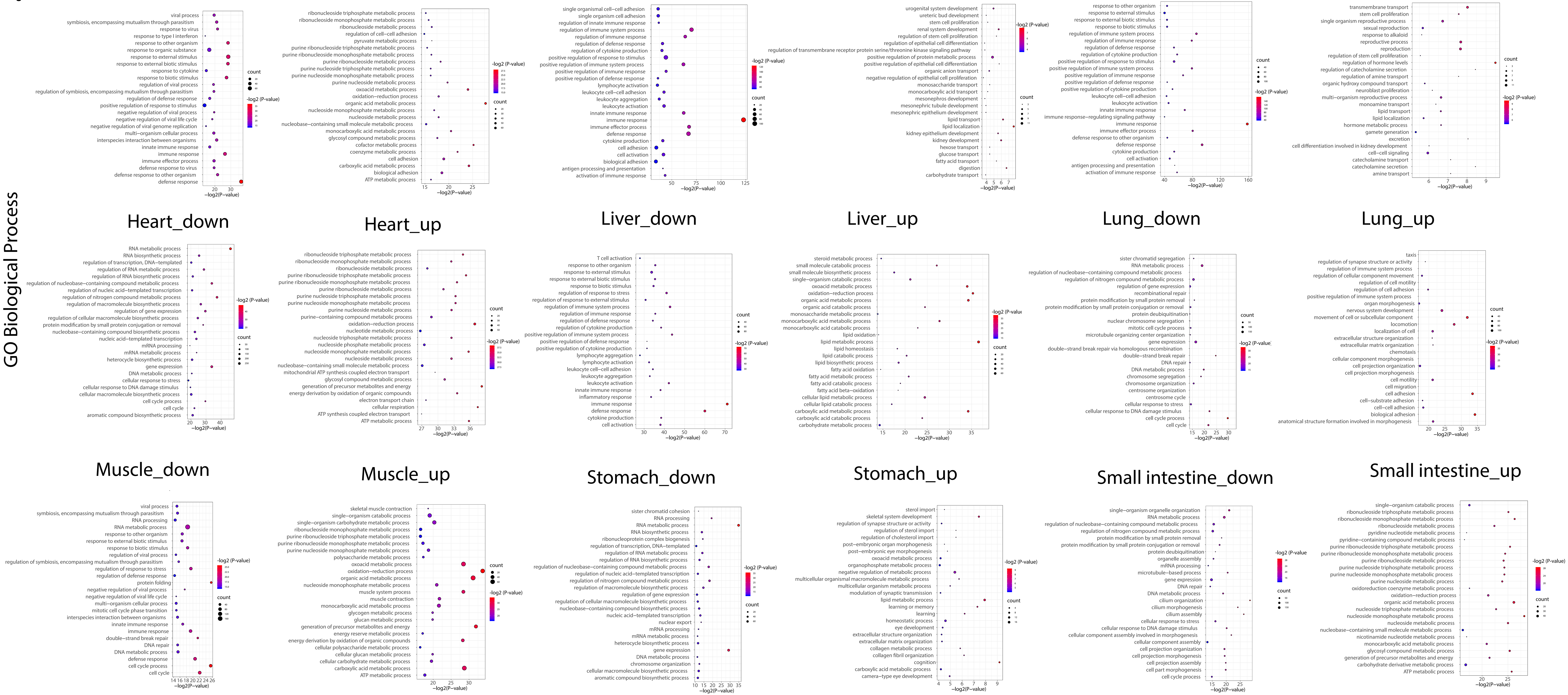
GO Biological Process
Adipose_down
viral process
symbiosis, encompassing mutualism through parasitism
response to virus
response to type I interferon
response to other organism
response to organic substance
response to external stimulus
response to external biotic stimulus
response to cytokine
response to biotic stimulus
regulation of viral process
regulation of symbiosis, encompassing mutualism through parasitism
regulation of defense response
positive regulation of response to stimulus
negative regulation of viral process
negative regulation of viral life cycle
negative regulation of viral genome replication
multi−organism cellular process
interspecies interaction between organisms
innate immune response
immune response
immune effector process
defense response to virus
defense response to other organism
defense response
−log2(P−value)
count
Adipose_up
ribonucleoside triphosphate metabolic process
ribonucleoside monophosphate metabolic process
ribonucleoside metabolic process
regulation of cell−cell adhesion
pyruvate metabolic process
purine ribonucleoside triphosphate metabolic process
purine ribonucleoside monophosphate metabolic process
purine ribonucleoside metabolic process
purine nucleoside triphosphate metabolic process
purine nucleoside monophosphate metabolic process
purine nucleoside metabolic process
oxoacid metabolic process
oxidation−reduction process
organic acid metabolic process
nucleoside monophosphate metabolic process
nucleoside metabolic process
nucleobase−containing small molecule metabolic process
monocarboxylic acid metabolic process
glycosyl compound metabolic process
cofactor metabolic process
coenzyme metabolic process
cell adhesion
carboxylic acid metabolic process
biological adhesion
ATP metabolic process
Cerebellum_down
single organismal cell−cell adhesion
single organism cell adhesion
regulation of innate immune response
regulation of immune system process
regulation of immune response
regulation of defense response
regulation of cytokine production
positive regulation of response to stimulus
positive regulation of immune system process
positive regulation of immune response
positive regulation of defense response
lymphocyte activation
leukocyte cell−cell adhesion
leukocyte aggregation
leukocyte activation
innate immune response
immune response
immune effector process
defense response
cytokine production
cell adhesion
cell activation
biological adhesion
antigen processing and presentation
activation of immune response
Cerebellum_up
urogenital system development
ureteric bud development
stem cell proliferation
renal system development
regulation of stem cell proliferation
regulation of epithelial cell differentiation
regulation of transmembrane receptor protein serine/threonine kinase signaling pathway
positive regulation of protein metabolic process
positive regulation of epithelial cell differentiation
organic anion transport
negative regulation of epithelial cell differentiation
monosaccharide transport
monocarboxylic acid transport
mesonephros development
mesonephric tubule development
mesonephric epithelium development
lipid transport
lipid localization
kidney epithelium development
kidney development
hexose transport
glucose transport
fatty acid transport
digestion
carbohydrate transport
Cerebrum_down
response to other organism
response to external stimulus
response to external biotic stimulus
response to biotic stimulus
regulation of immune system process
regulation of immune response
regulation of defense response
regulation of cytokine production
positive regulation of response to stimulus
positive regulation of immune system process
positive regulation of immune response
positive regulation of defense response
positive regulation of cytokine production
leukocyte cell−cell adhesion
leukocyte activation
innate immune response
immune response−regulating signaling pathway
immune response
immune effector process
defense response to other organism
defense response
cytokine production
cell activation
antigen processing and presentation
activation of immune response
Cerebrum_up
transmembrane transport
stem cell proliferation
single organism reproductive process
sexual reproduction
response to alkaloid
reproductive process
reproduction
regulation of stem cell proliferation
regulation of hormone levels
regulation of catecholamine secretion
regulation of amine transport
organic hydroxy compound transport
neuroblast proliferation
multi−organism reproductive process
monoamine transport
lipid transport
lipid localization
hormone metabolic process
gamete generation
excretion
cell differentiation involved in kidney development
cell−cell signaling
catecholamine transport
catecholamine secretion
amine transport
Heart_down
RNA metabolic process
RNA biosynthetic process
regulation of transcription, DNA−templated
regulation of RNA metabolic process
regulation of RNA biosynthetic process
regulation of nucleobase−containing compound metabolic process
regulation of nucleic acid−templated transcription
regulation of nitrogen compound metabolic process
regulation of macromolecule biosynthetic process
regulation of gene expression
regulation of cellular macromolecule biosynthetic process
protein modification by small protein conjugation or removal
nucleobase−containing compound biosynthetic process
nucleic acid−templated transcription
mRNA processing
mRNA metabolic process
heterocycle biosynthetic process
gene expression
DNA metabolic process
cellular response to stress
cellular response to DNA damage stimulus
cellular macromolecule biosynthetic process
cell cycle process
cell cycle
aromatic compound biosynthetic process
Heart_up
ribonucleoside triphosphate metabolic process
ribonucleoside monophosphate metabolic process
ribonucleoside metabolic process
purine ribonucleoside triphosphate metabolic process
purine ribonucleoside monophosphate metabolic process
purine ribonucleoside metabolic process
purine nucleoside triphosphate metabolic process
purine nucleoside monophosphate metabolic process
purine nucleoside metabolic process
purine−containing compound metabolic process
oxidation−reduction process
nucleotide metabolic process
nucleoside triphosphate metabolic process
nucleoside phosphate metabolic process
nucleoside monophosphate metabolic process
nucleoside metabolic process
nucleobase−containing small molecule metabolic process
mitochondrial ATP synthesis coupled electron transport
glycosyl compound metabolic process
generation of precursor metabolites and energy
energy derivation by oxidation of organic compounds
electron transport chain
cellular respiration
ATP synthesis coupled electron transport
ATP metabolic process
Liver_down
T cell activation
response to other organism
response to external stimulus
response to external biotic stimulus
response to biotic stimulus
regulation of response to stress
regulation of response to external stimulus
regulation of immune system process
regulation of immune response
regulation of defense response
regulation of cytokine production
positive regulation of immune system process
positive regulation of defense response
positive regulation of cytokine production
lymphocyte aggregation
lymphocyte activation
leukocyte cell−cell adhesion
leukocyte aggregation
leukocyte activation
innate immune response
inflammatory response
immune response
defense response
cytokine production
cell activation
Liver_up
steroid metabolic process
small molecule catabolic process
small molecule biosynthetic process
single−organism catabolic process
oxoacid metabolic process
oxidation−reduction process
organic acid metabolic process
organic acid catabolic process
monosaccharide metabolic process
monocarboxylic acid metabolic process
monocarboxylic acid catabolic process
lipid oxidation
lipid metabolic process
lipid homeostasis
lipid catabolic process
lipid biosynthetic process
fatty acid oxidation
fatty acid metabolic process
fatty acid catabolic process
fatty acid beta−oxidation
cellular lipid metabolic process
cellular lipid catabolic process
carboxylic acid metabolic process
carboxylic acid catabolic process
carbohydrate metabolic process
Lung_down
sister chromatid segregation
RNA metabolic process
regulation of nucleobase−containing compound metabolic process
regulation of nitrogen compound metabolic process
regulation of gene expression
recombinational repair
protein modification by small protein removal
protein modification by small protein conjugation or removal
protein deubiquitination
nuclear chromosome segregation
mitotic cell cycle process
microtubule organizing center organization
gene expression
double−strand break repair via homologous recombination
double−strand break repair
DNA repair
DNA metabolic process
chromosome segregation
chromosome organization
centrosome organization
centrosome cycle
cellular response to stress
cellular response to DNA damage stimulus
cell cycle process
cell cycle
Lung_up
taxis
regulation of synapse structure or activity
regulation of immune system process
regulation of cellular component movement
regulation of cell motility
regulation of cell adhesion
positive regulation of immune system process
organ morphogenesis
nervous system development
movement of cell or subcellular component
locomotion
localization of cell
extracellular structure organization
extracellular matrix organization
chemotaxis
cellular component morphogenesis
cell projection organization
cell projection morphogenesis
cell motility
cell migration
cell adhesion
cell−substrate adhesion
cell−cell adhesion
biological adhesion
anatomical structure formation involved in morphogenesis
Muscle_down
viral process
symbiosis, encompassing mutualism through parasitism
RNA processing
RNA metabolic process
response to other organism
response to external biotic stimulus
response to biotic stimulus
regulation of viral process
regulation of symbiosis, encompassing mutualism through parasitism
regulation of response to stress
regulation of defense response
protein folding
negative regulation of viral process
negative regulation of viral life cycle
multi−organism cellular process
mitotic cell cycle phase transition
interspecies interaction between organisms
innate immune response
immune response
double−strand break repair
DNA repair
DNA metabolic process
defense response
cell cycle process
cell cycle
Muscle_up
skeletal muscle contraction
single−organism catabolic process
single−organism carbohydrate metabolic process
ribonucleoside monophosphate metabolic process
purine ribonucleoside monophosphate metabolic process
purine ribonucleoside triphosphate metabolic process
purine nucleoside monophosphate metabolic process
purine nucleoside metabolic process
polysaccharide metabolic process
oxoacid metabolic process
oxidation−reduction process
organic acid metabolic process
nucleoside monophosphate metabolic process
muscle system process
muscle contraction
monocarboxylic acid metabolic process
glycogen metabolic process
glucan metabolic process
generation of precursor metabolites and energy
energy reserve metabolic process
energy derivation by oxidation of organic compounds
cellular polysaccharide metabolic process
cellular glucan metabolic process
cellular carbohydrate metabolic process
carboxylic acid metabolic process
ATP metabolic process
Stomach_down
sister chromatid cohesion
RNA processing
RNA metabolic process
RNA biosynthetic process
ribonucleoprotein complex biogenesis
regulation of transcription, DNA−templated
regulation of RNA metabolic process
regulation of RNA biosynthetic process
regulation of nucleobase−containing compound metabolic process
regulation of nucleic acid−templated transcription
regulation of nitrogen compound metabolic process
regulation of macromolecule biosynthetic process
regulation of gene expression
regulation of cellular macromolecule biosynthetic process
nucleobase−containing compound biosynthetic process
nucleic acid−templated transcription
nuclear export
mRNA processing
mRNA metabolic process
heterocycle biosynthetic process
gene expression
DNA metabolic process
chromosome organization
cellular macromolecule biosynthetic process
aromatic compound biosynthetic process
Stomach_up
sterol import
skeletal system development
regulation of synapse structure or activity
regulation of sterol import
regulation of cholesterol import
post−embryonic organ morphogenesis
post−embryonic eye morphogenesis
oxoacid metabolic process
organophosphate metabolic process
negative regulation of metabolic process
multicellular organismal macromolecule metabolic process
multicellular organism metabolic process
modulation of synaptic transmission
lipid metabolic process
learning or memory
learning
homeostatic process
eye development
extracellular structure organization
extracellular matrix organization
collagen metabolic process
collagen fibril organization
cognition
carboxylic acid metabolic process
camera−type eye development
Small intestine_down
single−organism organelle organization
RNA metabolic process
regulation of nucleobase−containing compound metabolic process
regulation of nitrogen compound metabolic process
protein modification by small protein removal
protein modification by small protein conjugation or removal
protein deubiquitination
organelle assembly
mRNA processing
microtubule−based process
gene expression
DNA repair
DNA metabolic process
cilium organization
cilium morphogenesis
cilium assembly
cellular response to stress
cellular response to DNA damage stimulus
cellular component assembly involved in morphogenesis
cellular component assembly
cell projection organization
cell projection morphogenesis
cell projection assembly
cell part morphogenesis
cell cycle process
Small intestine_up
single−organism catabolic process
ribonucleoside triphosphate metabolic process
ribonucleoside monophosphate metabolic process
ribonucleoside metabolic process
pyridine nucleotide metabolic process
pyridine−containing compound metabolic process
purine ribonucleoside triphosphate metabolic process
purine ribonucleoside monophosphate metabolic process
purine ribonucleoside metabolic process
purine nucleoside triphosphate metabolic process
purine nucleoside monophosphate metabolic process
purine nucleoside metabolic process
oxidoreduction coenzyme metabolic process
oxidation−reduction process
organic acid metabolic process
nucleoside triphosphate metabolic process
nucleoside monophosphate metabolic process
nucleoside metabolic process
nucleobase−containing small molecule metabolic process
nicotinamide nucleotide metabolic process
monocarboxylic acid metabolic process
glycosyl compound metabolic process
generation of precursor metabolites and energy
carbohydrate derivative metabolic process
ATP metabolic process
−log2(P−value)
count

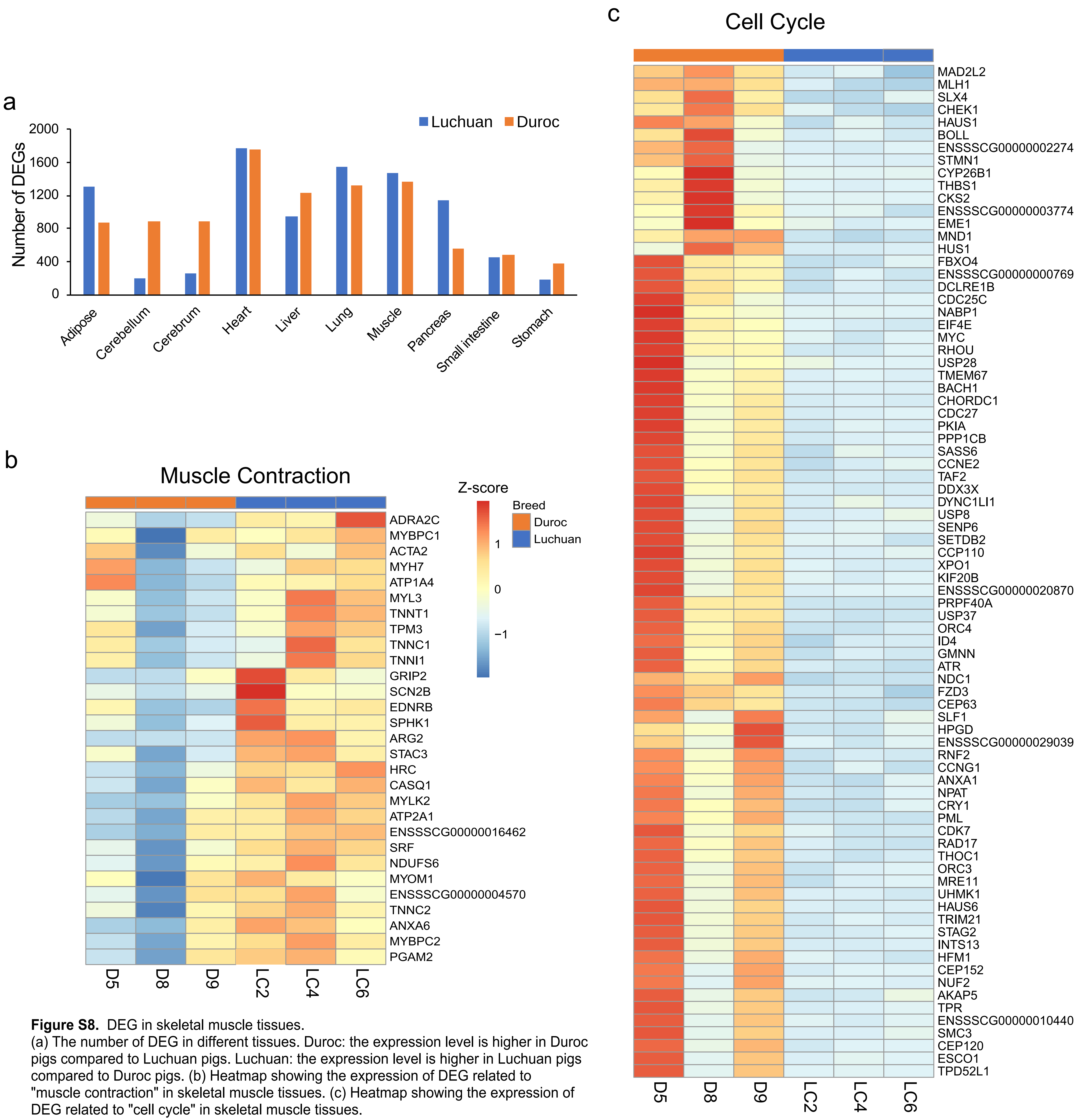

Figure S8. DEG in skeletal muscle tissues.
(a) The number of DEG in different tissues. Duroc: the expression level is higher in Duroc pigs compared to Luchuan pigs. Luchuan: the expression level is higher in Luchuan pigs compared to Duroc pigs. (b) Heatmap showing the expression of DEG related to "muscle contraction" in skeletal muscle tissues. (c) Heatmap showing the expression of DEG related to "cell cycle" in skeletal muscle tissues.

Figure S9. Frequencies of the Luchuan promoter allele across different pig breeds. DUR: Duroc pig; LW: Large White pig; LD: Landrace pig; YM: Yucatan miniature pig ; YNT: Tibetan pig (Yunnan); TC: Tongcheng pig; TT: Tibetan pig (Qinghai-Tibetan); HTDE: Hetaodaer pig; SCT: Tibetan pig (Sichuan); WZS: Wuzhishan pig; BMX: Bamaxiang pig; EHL: Erhualian pig; LUC: Luchuan pig; LWH: Laiwu black pig; MIN: Min pig (Shandong).

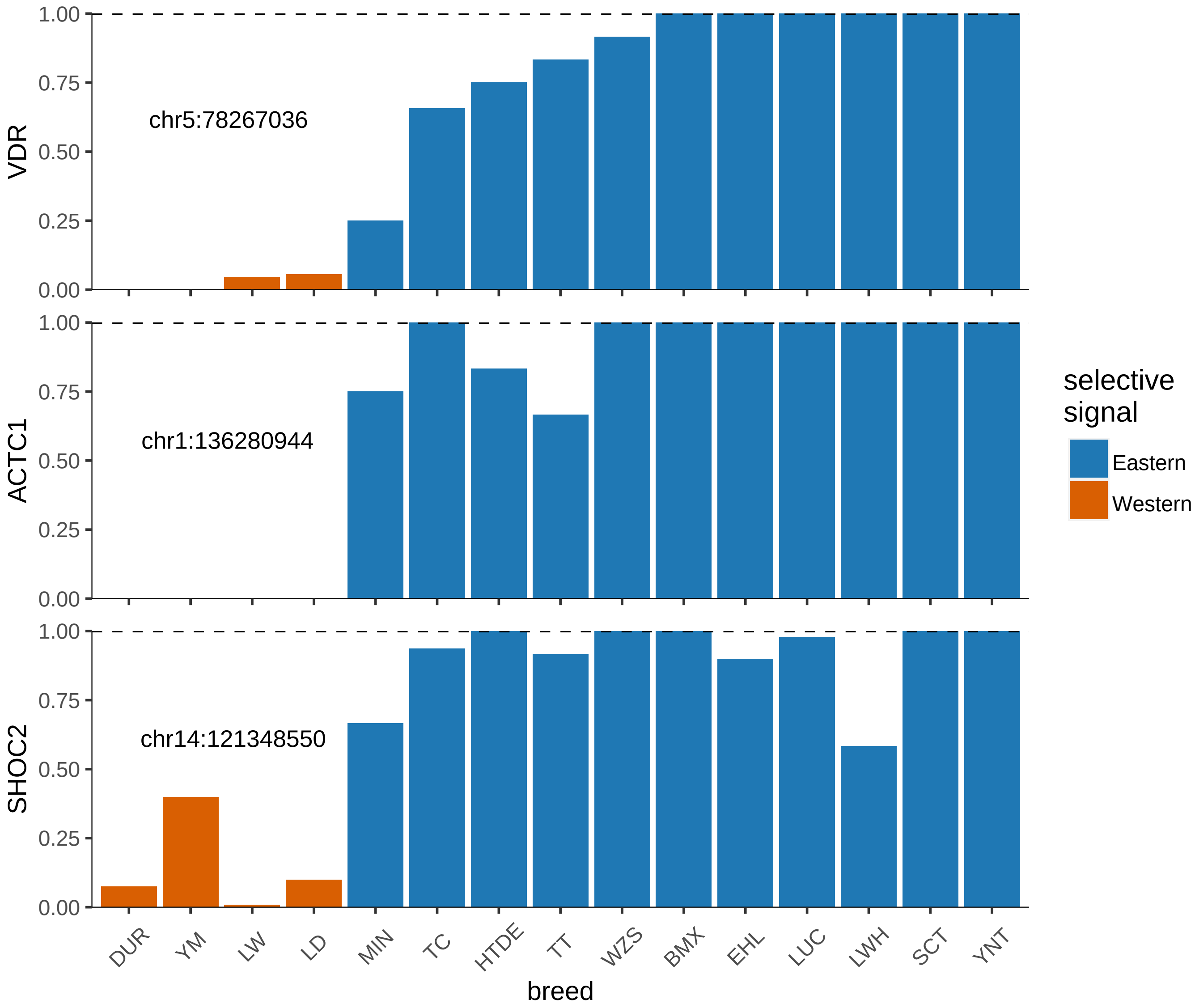

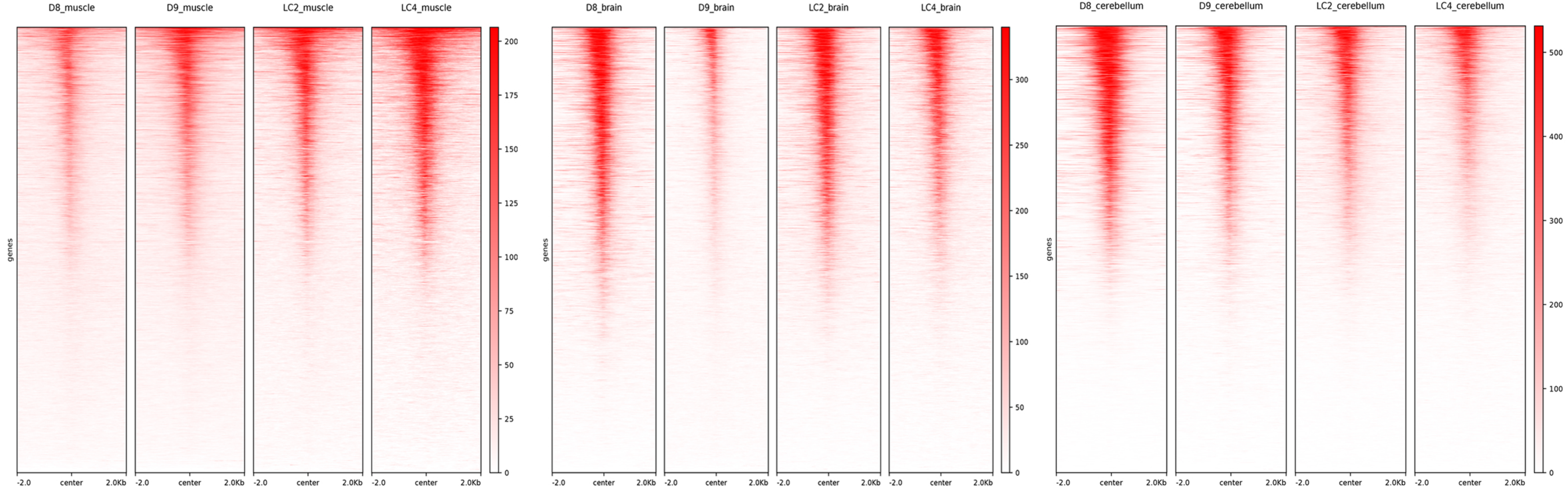

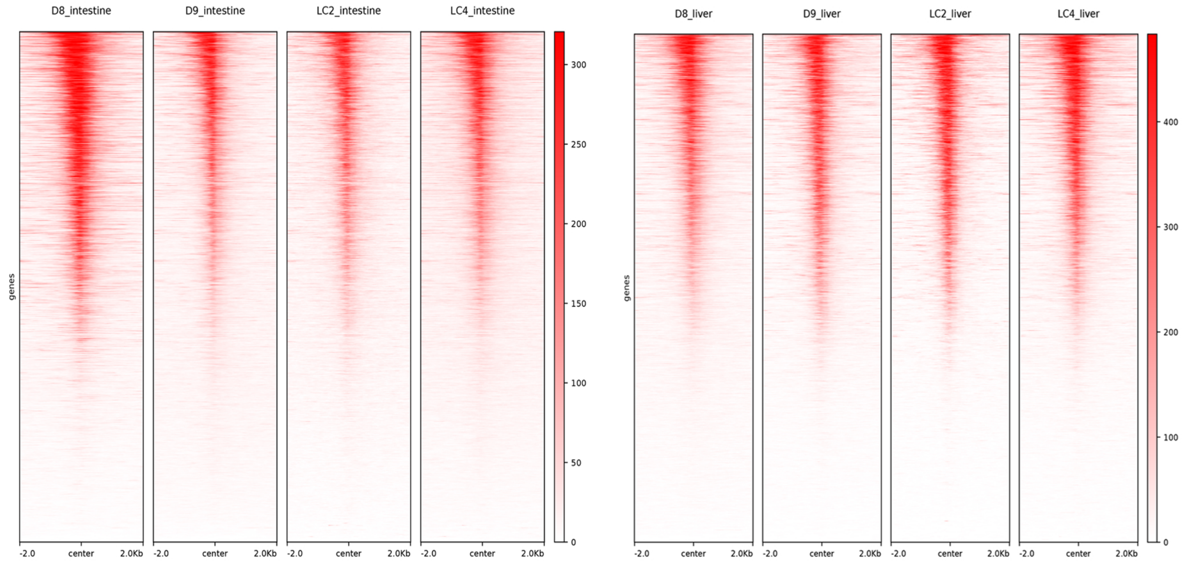

Figure S10. QC of ATAC-seq peaks. Heatmap of reads density around TSS for five tissues.

Figure S11. Muscle fiber differences between Duroc and Luchuan pigs. Proportions of slow muscle fiber.

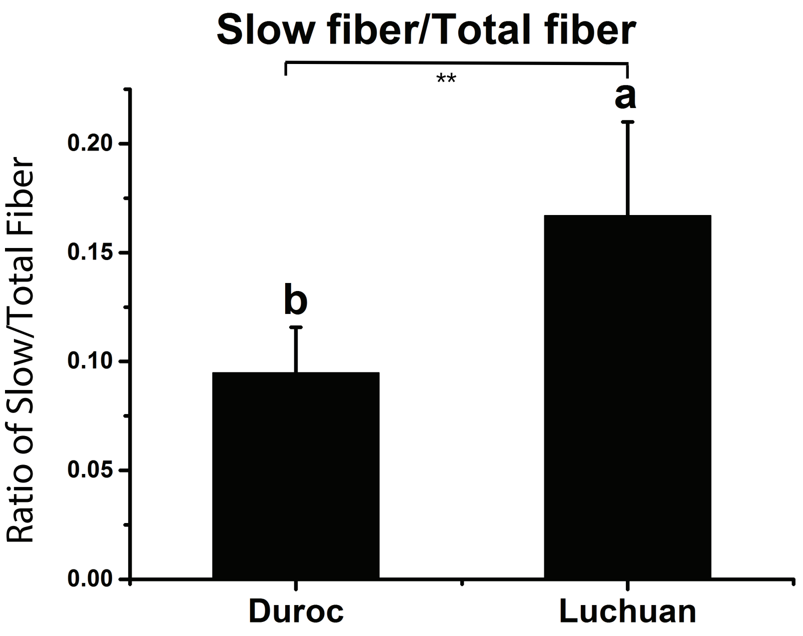

Figure S12. Knockdown and overexpression efficiency of *Tnnc1* in C2C12 cells and in vivo. (a) *Tnnc1* expression after siRNA knockdown in C2C12 cells. (b) *Tnnc1* expression after overexpression in C2C12 cells. (c) *Tnnc1* expression after in vivo siRNA knockdown in mouse skeletal muscle.

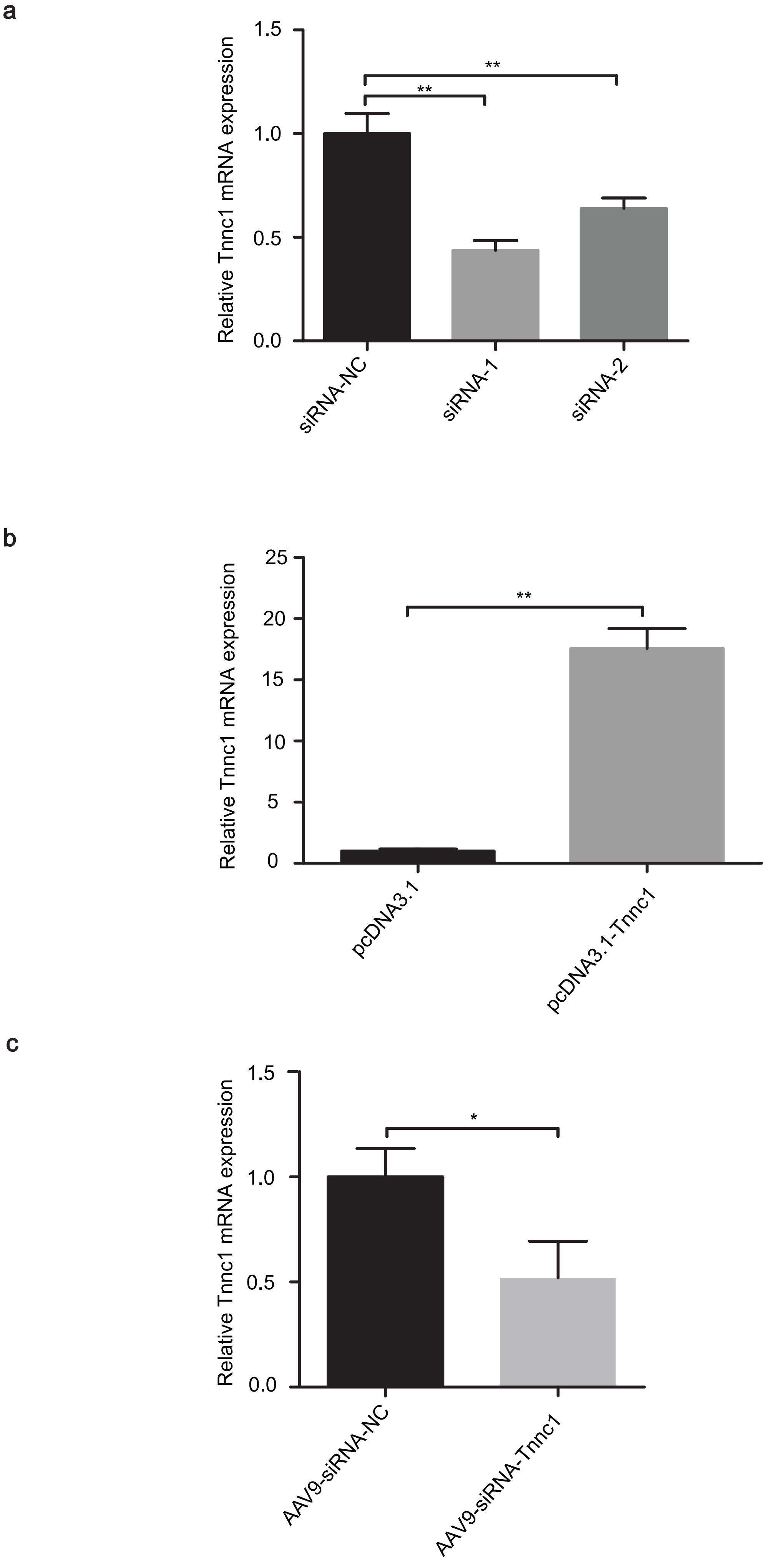

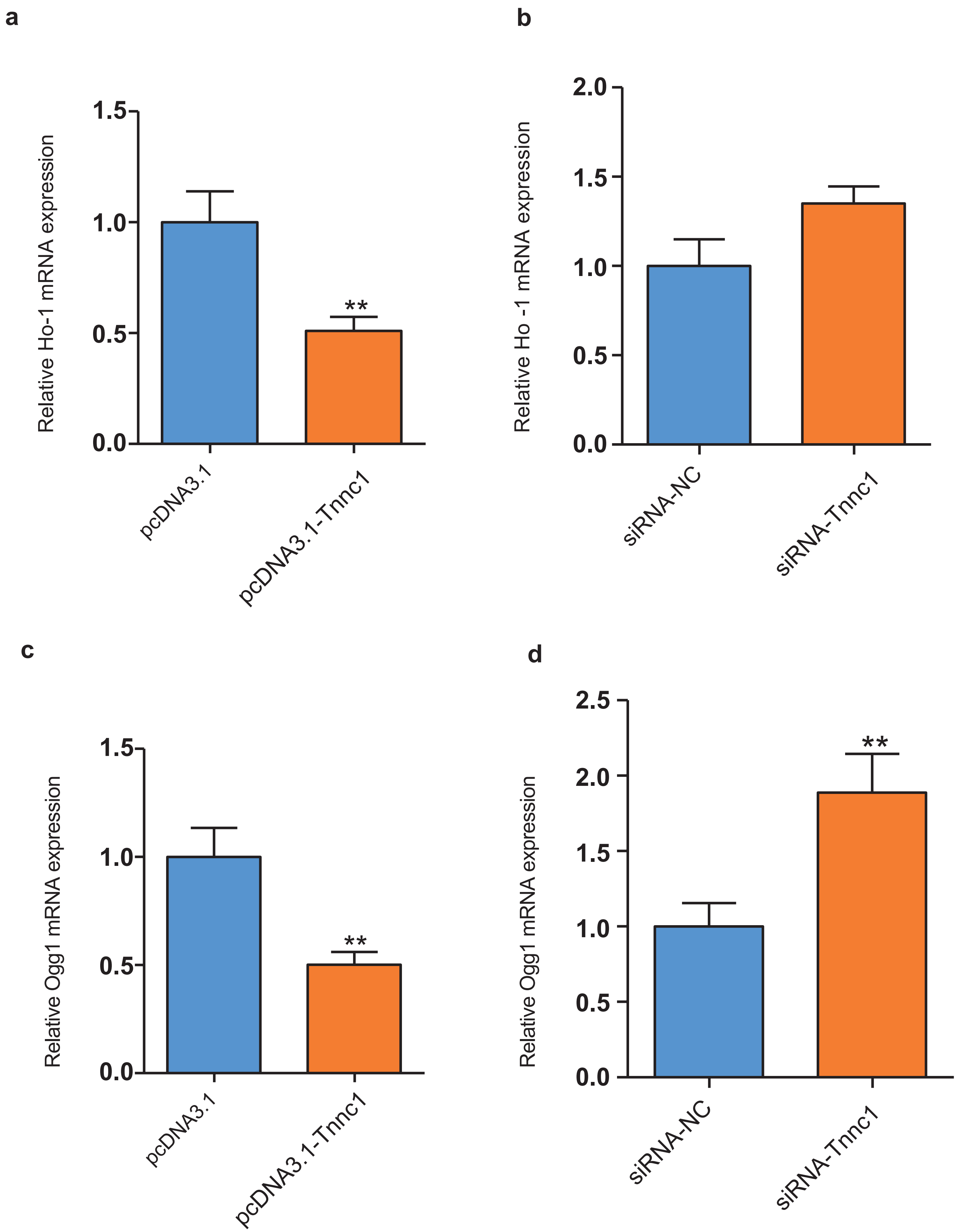

Figure S13. (a) Expression of *Ho-1* after overexpression of *Tnnc1*.(b) Expression of *Ho-1* after siRNA konckdown of *Tnnc1*. (c) Expression of *Ogg1* afteroverexpression of *Tnnc1*. (d) Expression of *Ogg1* after siRNA knockdown of *Tnnc1*.

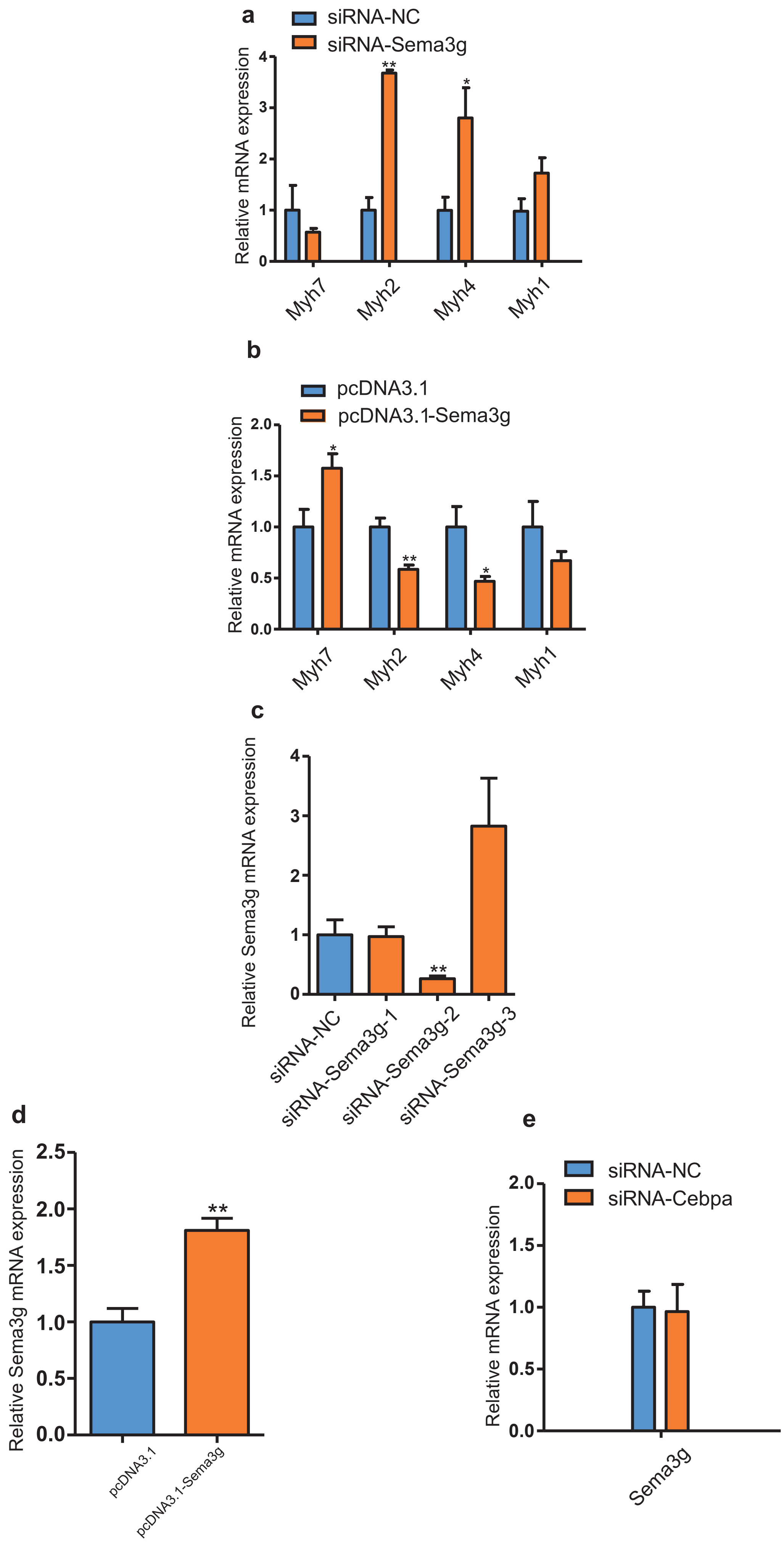

Figure S14. (a). Expression of slow muscle (*Myh7*) and fast muscle (*Myh2*, *Myh4*, *Myh1*) markers after siRNA knockdown of *Sema3g* in mouse C2C12 cells (b). Measuring the same markers as (a) after overexpression of *Sema3g* in mouse C2C12 cells. (c). Choosing siRNAs with the best knockdown efficiency. (d). Evaluation of the overexpression efficiency. (e). Comparing the expression level of *Sema3g* before and after knocking down *Cebpa*. * represents p value < 0.05. ** represents p value < 0.01.
